# High‐Efficiency Carriers’ Separation Strategy Based Ultrasmall‐Bandgap CuWO_4_ Sono‐Enhances GSH Antagonism for Cuproptosis Cascade Immunotherapy

**DOI:** 10.1002/advs.202500576

**Published:** 2025-05-21

**Authors:** Lichao Zhu, Zhisheng Guo, Yu Luo, Haiyan Huang, Kexin Zhang, Bingbing Duan, Renmiao Peng, Haochen Yao, Chao Liang, Kaiyang Wang

**Affiliations:** ^1^ Shanghai Engineering Research Center of Pharmaceutical Intelligent Equipment Shanghai Frontiers Science Research Center for Druggability of Cardiovascular Non‐coding RNA Institute for Frontier Medical Technology School of Chemistry and Chemical Engineering Shanghai University of Engineering Science Shanghai 201620 P. R. China; ^2^ Department of Urology The First Affiliated Hospital of Nanjing Medical University and Jiangsu Province Hospital Nanjing 210029 P. R. China; ^3^ Hepatobiliary and Pancreatic Surgery Department General Surgery Center First Hospital of Jilin University No.1 Xinmin Street Changchun Jilin 130021 P. R. China

**Keywords:** cuproptosis, mitochondrial dysfunction, nanoparticles, oxidative stress

## Abstract

The spatiotemporal sequential treatment strategy of promoting rapid separation of charge carriers, amplifying oxidative stress, increasing the low content of intracellular Cu, enhancing cuproptosis, and cascading activation of immunotherapy is considered one of the most effective techniques for improving the comprehensive therapy of tumors. Herein, copper tungstate (CuWO₄, CWO) nanoparticles with ultrasmall bandgap (1.71 eV) is developed as both piezoelectric‐catalysis agents and copper nanocarriers for synergistic sono‐enhanced cuproptosis. Owing to the unique bandgap microstructure, exposure to ultrasound (US) significantly increase the generation of reactive oxygen species (ROS) and the release of Cu^2+^ from CWO. Additionally, ≈60% of glutathione (GSH) and nicotinamide adenine dinucleotide phosphate (NADPH) are consumed in situ, leading to oxidative stress, ferroptosis, and cuproptosis in cancer cells. This cascading approach induces substantial mitochondrial dysfunction and the release of damage‐associated molecular patterns (DAMPs), which promotes immunogenic cell death (ICD) and augments antitumor immunity. Both in vitro and in vivo studies have shown that this sono‐enhanced cuproptosis‐based therapy could effectively suppress tumor growth. Overall, this study investigates a novel Structure‐Function therapeutic approach that combines piezoelectric catalysis, ferroptosis, cuproptosis, and cascade activation of immune regulation, opening up new possibilities for addressing the challenges associated with conventional cuproptosis therapy.

## Graphical abstract

This study prepares copper tungstate (CuWO_4_, CWO) nanoparticles for synergistic sono‐enhanced cuproptosis‐based cancer therapy. With an ultrasmall bandgap (1.71 eV), CWO facilitates efficient charge carrier separation under ultrasound, enhancing the generation of reactive oxygen species and the controlled release of Cu^2+^ ions cascade amplify oxidative stress. Additionally, CWO‐mediated Cu^2+^ release triggers cuproptosis, inducing significant mitochondrial dysfunction and activates antitumor immunity.

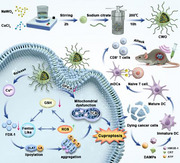

## 1 Introduction

Sonodynamic therapy (SDT), a rapidly emerging non‐invasive cancer treatment modality, harnesses ultrasound (US) to activate sonosensitizers, producing reactive oxygen species (ROS) that induce oxidative stress and subsequent tumor cell death. Thanks to the US's superior penetration capability of over 10 cm, it enables the precise treatment of tumors located at depths beyond the reach of conventional light‐based therapies.^[^ ^1^, ^2^, ^3^, ^4^, ^5^ ^]^ However, the application of SDT in cancer treatment is often limited by insufficient production of ROS.^[^ ^6^, ^7^, ^8^, ^9^ ^]^ Organic sonosensitizers, such as porphyrins, face challenges with low enrichment, high phototoxicity, and poor solubility. In contrast, inorganic sonosensitizers like TiO_2_ typically exhibit lower US coefficients and faster electron‐hole recombination.^[^ ^10^, ^11^ ^]^ Therefore, highly efficient sonosensitizers are desired for optimal SDT applications.

Intriguingly, piezoelectric sonosensitizers show great potential in SDT owing to their unique acoustoelectric characteristics. When exposed to US irradiation, the relative movement of positive and negative ions within the crystal structure of these materials hinders the direct recombination of charge centers, leading to the generation of an internal electric field and piezoelectric potential. This potential effectively promotes electron‐hole separation and reduces recombination. Consequently, the separated electron‐hole pairs migrate to the surface to produce ROS.^[^ ^12^, ^13^, ^14^, ^15^, ^16^ ^]^ However, the ROS generation capability of piezoelectric sonosensitizers is constrained by a high activation energy barrier, which is attributed to their suboptimal bandgap. Therefore, there is a need for novel piezoelectric sonosensitizers with a small bandgap. Inspired by recent advancements in cuproptosis and immunotherapy, the integration of piezoelectric sonosensitizers with these modalities could potentially enhance the overall efficiency and comprehensiveness of tumor treatment.^[^ ^17^, ^18^, ^19^ ^]^

Unlike traditional programmed cell death pathways, cuproptosis is Cu and mitochondria‐respiration dependent.^[^ ^20^, ^21^, ^22^, ^23^ ^]^ By direct binding Cu ions to lipoylated components of the tricarboxylic acid (TCA) cycle, cuproptosis results in the aggregation of lipoylated proteins and the degradation of Fe‐S cluster proteins, eventually triggering protein toxicity stress and cell death.^[^ ^24^, ^25^, ^26^ ^]^ However, the concentration of copper ions in tumor tissues is typically insufficient to achieve therapeutic effects, and excess copper is expelled from cells by copper transporters ATP7A and ATP7B, preventing harmful intracellular accumulation.^[^ ^27^, ^28^, ^29^ ^]^ Therefore, the development of smart delivery systems to increase copper concentration and enhance copper release at the tumor site is highly desirable to harness copper‐induced cell death as a potential cancer treatment.

**Scheme 1:**
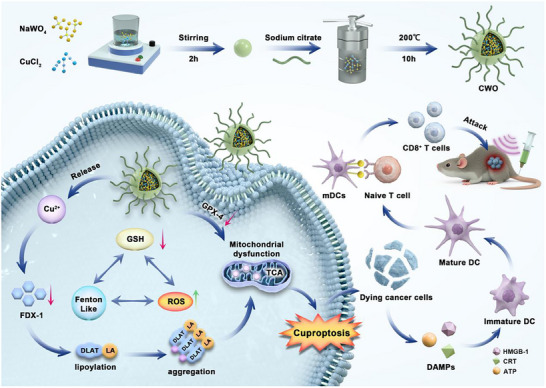
Synthesis of CWO and oxidative stress amplification‐enhanced cuproptosis‐based therapy for pancreatic cancer.

By combining US with copper‐based piezoelectric sonosensitizers, the sono‐enhanced cuproptosis‐based synergistic therapy could offer new opportunities for effective and safe cancer treatment. Herein, copper tungstate (CuWO_4_, referred to as CWO) nanoparticles were synthesized using a one‐pot hydrothermal method. As illustrated in **Scheme** **1**, once injected in vivo, CWO accumulates efficiently at the tumor site through the enhanced permeability and retention (EPR) effect. Under US irradiation, mechanical deformation of CWO triggers the piezoelectric effect, generating an internal electric field. Simultaneously, the narrow bandgap of CWO facilitates bending of its valence and conduction bands under the influence of this electric field. This piezoelectric field effectively suppresses electron‐hole recombination, prolonging the lifetime of charge carriers and enabling holes to directly oxidize water molecules, thus producing hydroxyl radicals (•OH). Consequently, these generated •OH induce oxidative stress in cancer cells. Furthermore, it was found that CWO could deplete glutathione (GSH) and nicotinamide adenine dinucleotide phosphate (NADPH), further enhancing oxidative stress and inducing ferroptosis. More importantly, under the acidic conditions within the tumor and the influence of US, CWO releases Cu ions, which modulate the DLAT, leading to cuproptosis in tumor cells. These synergistic effects of induced oxidative stress, ferroptosis, and cuproptosis ultimately lead to mitochondrial dysfunction and release of damage‐associated molecular patterns (DAMPs), resulting in immunogenic cell death (ICD) and enhancing cancer immunotherapy. CWO is effective and safe for inhibiting cancer both in vitro and in vivo. This study offers a promising approach for developing cancer therapies targeting mitochondrial dysfunction and leveraging ferroptosis and cuproptosis mechanisms.

## 2 Results and Discussion

**Figure 1:**
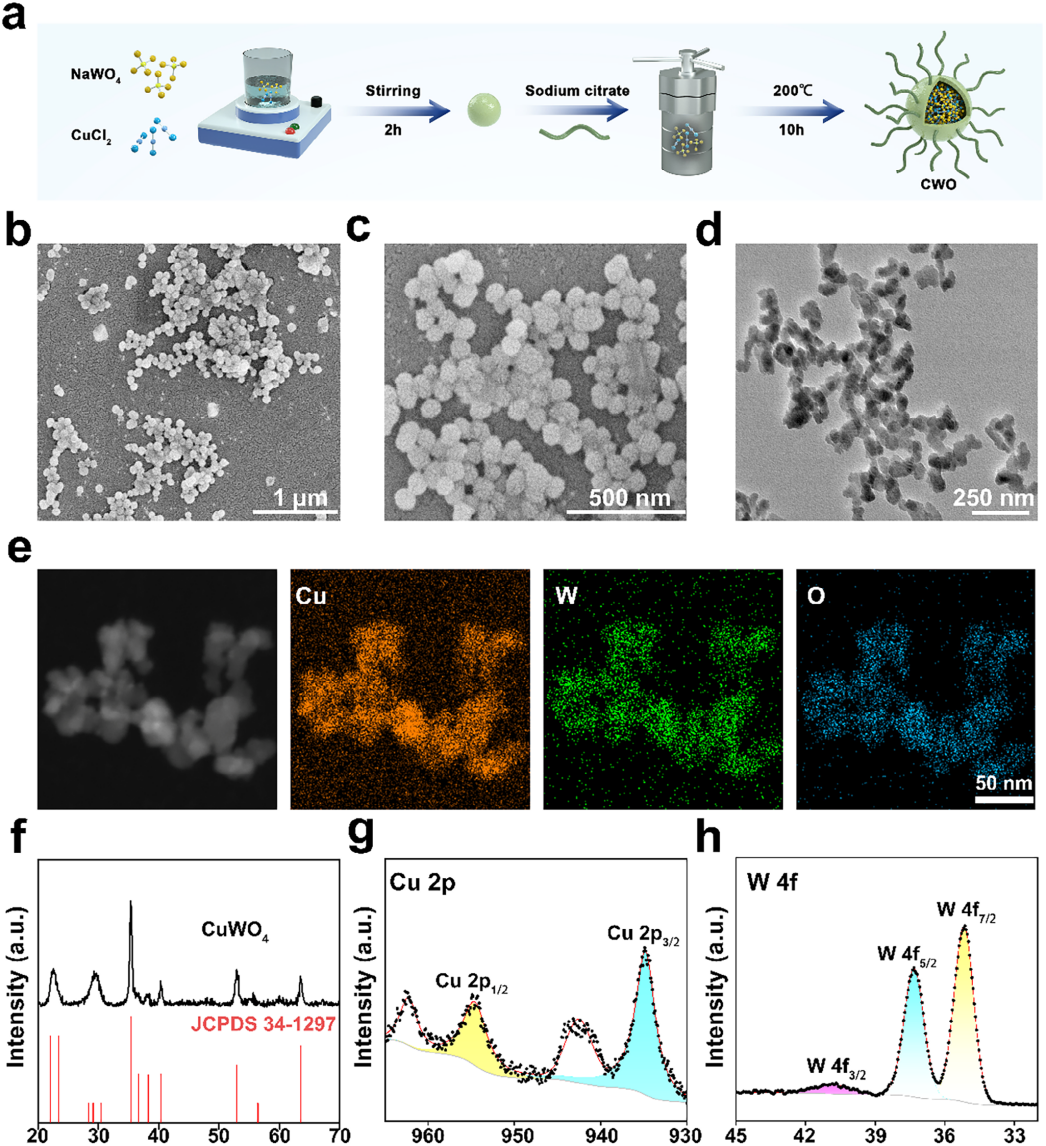
Characterization of CWO. a) Synthesis of CWO. b) and c) SEM image, and d) TEM images of CWO. e). Elemental mapping for Cu, W, and O. f) XRD pattern of CWO. High‐resolution XPS spectra of: g) Cu and h) W in CWO.

A one‐pot hydrothermal method was employed to synthesize CWO nanoparticles, as illustrated in **Figure** **1**a. Scanning electron microscopy (SEM) images (Figure 1b,c) and transmission electron microscopy (TEM) images (Figure 1d) showed that CWO has a uniform spherical structure. Additionally, low‐magnification TEM imaging (Figure S1, Supporting Information) demonstrated that the synthesized CWO nanoparticles were well‐dispersed without significant aggregation. Furthermore, the size distribution of 110 nanoparticles measured from SEM images revealed an average particle size of ≈59 nm, with particle sizes ranging from 37 to 101 nm (Figure S2, Supporting Information). Subsequently, atomic force microscopy (AFM) was employed to examine the size and morphology of the CWO. The planar AFM image (Figure S3, Supporting Information) showed that CWO features a spherical structure with a size of 41.5 nm. Elemental mapping further confirmed a uniform distribution of Cu, O, and W throughout the nanoparticles, indicative of the successful synthesis of CWO (Figure 1e). Furthermore, X‐ray diffraction (XRD) analysis confirmed the presence of distinctive peaks corresponding to Cu_2_WO_4_(OH)_2_ (JCPDS NO. 34‐1297), as shown in Figure 1f, which verifies the crystalline nature of the synthesized product. X‐ray photoelectron spectroscopy (XPS) further verified the presence of Cu, O, and W in CWO (Figure S4, Supporting Information). High‐resolution XPS analysis provided more detailed insights into the valence states of the copper and tungsten elements. The Cu 2p spectrum, fitted using Gaussian profiles, showed two distinct peaks at 955.2 and 935.2 eV, corresponding to the Cu 2p_1/2_ and Cu 2p_3/2_ orbitals, respectively, confirming the Cu^2+^ oxidation state (Figure 1g). The W 4f spectrum displayed peaks at 40.9, 37.6, and 35.6 eV, which are consistent with the binding energies of W 4f_3/2_, W 4f_5/2_, and W 4f_7/2_, respectively, indicating the presence of the W^6+^ oxidation state (Figure 1h). Together, these detailed analyses convincingly demonstrate the successful fabrication of the CWO nanoparticles.

The size and stability of the CWO nanoparticles were thoroughly analyzed. Dynamic light scattering (DLS) measurements indicated that the average particle sizes of CWO were ≈56 nm in DI water and 72 nm in a DMEM culture medium, with a polydispersity index (PDI) of 0.18 and 0.19, respectively (Figure S5, Supporting Information). These results indicate a relatively narrow size distribution, suggesting uniform particle dispersion in both media. Moreover, the zeta potential of the CWO nanoparticles was measured at −29 mV (Figure S6, Supporting Information), suggesting adequate electrostatic repulsion to prevent particle aggregation. Fourier‐transform infrared (FTIR) spectroscopy was employed to verify the successful surface modification of CWO with citric acid, as indicated by the characteristic peaks in the spectrum that correspond to citric acid (Figure S7, Supporting Information). Thanks to the modification of sodium citrate, the CWO showed excellent dispersibility and stability over 7 days of storage with no significant particle aggregation or size variation in either medium (Figure S8, Supporting Information). Additionally, a systematic evaluation under simulated physiological conditions (DMEM supplemented with 10% FBS and 1% Pen‐strep) demonstrated that CWO nanoparticles maintained excellent colloidal stability over a period of 7 days. These findings strongly support the stability of CWO nanoparticles in biological microenvironments, laying a solid foundation for their therapeutic applications.

**Figure 2:**
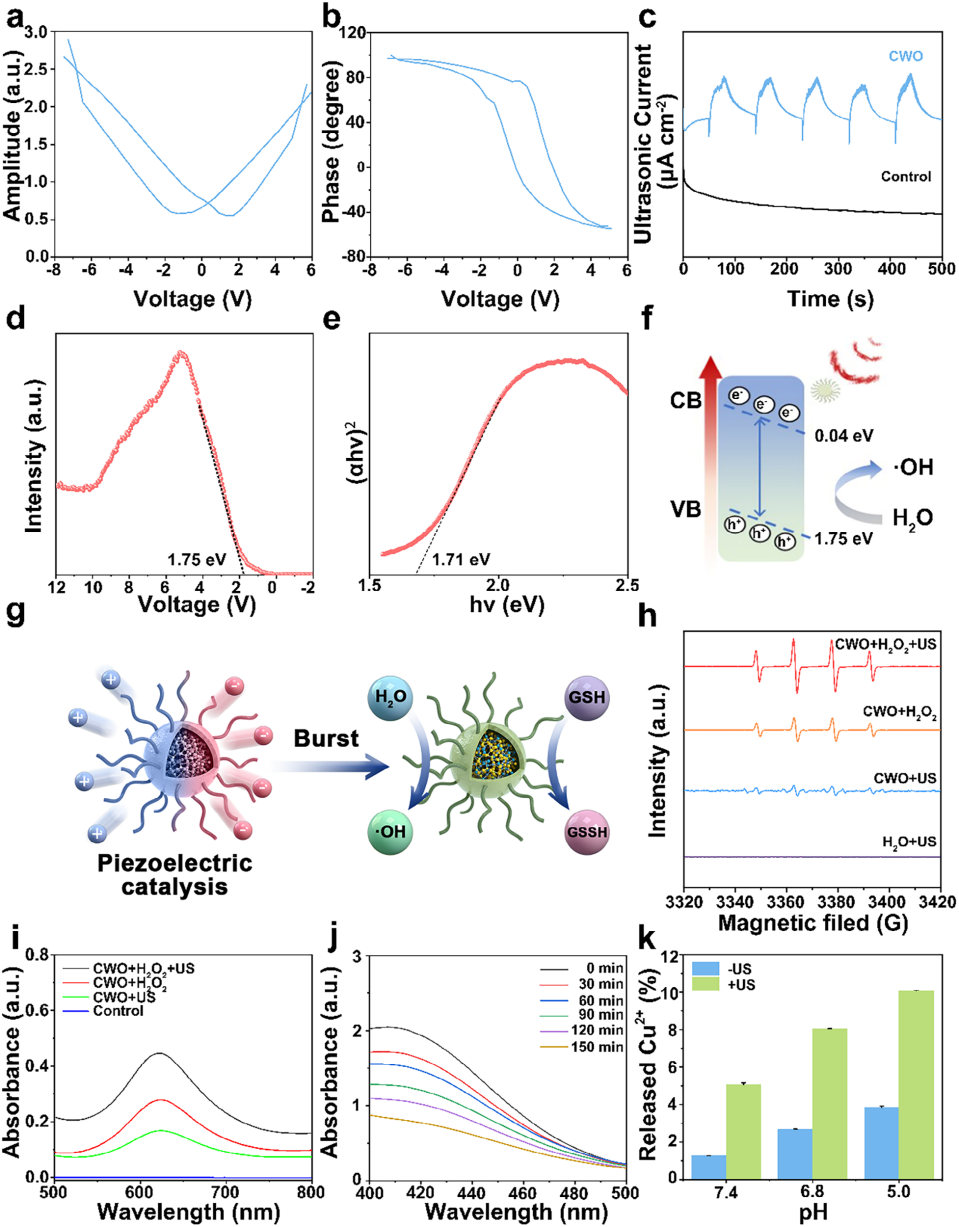
Piezoelectric performance of CWO. a) Butterfly curve and b) phase spectrum of CWO using PFM. c) Ultrasonic current density under US irradiation. d) VB spectra of CWO. e) Calculation of CWO band gap. f) The piezoelectric mechanism of CWO. g) Schematic illustration of TMB and DTNB probe. h). ESR spectra of CWO after various treatments. i) TMB oxidation of CWO after various treatments. j) Time‐dependent GSH depletion by CWO. k) Release of Cu^2+^ from CWO at varying pH and with or without US.

The application of US causes mechanical deformation, which induces a piezoelectric effect and generates an intrinsic electric field. This electric field promotes the transfer of electrons from the valence band (VB) to the conduction band (CB) and the formation of holes in the VB.^[^ ^6^, ^26^, ^27^ ^]^ To explore the piezoelectric mechanism of CWO under US irradiation, various characterizations were performed. First, the phase‐voltage response of CWO was characterized using typical piezoresponse force microscopy (PFM). Simultaneously, the phase image of CWO at an applied voltage of 10 V clearly demonstrated its piezoelectric properties. Besides the characteristic butterfly‐shaped amplitude–voltage curve, the PFM phase hysteresis loop exhibited a 180° phase shift when the direct‐current voltage varied from −10 V to +10 V (**Figure** **2**a,b). Moreover, the piezoelectric performance of CWO was evaluated under different pH conditions by calculating the piezoelectric coefficient (D33). Results showed that the D33 value of CWO was 10.9 pm V^−1^ at pH 5 and decreased to 4.9 pm V^−1^ at pH 7, whereas at pH 9, the piezoelectric properties of CWO became negligible, as evidenced by the unclosed hysteresis loop, making it impossible to calculate the D33 value accurately (Figure S9, Supporting Information). These findings clearly demonstrate that CWO exhibits significantly enhanced piezoelectric performance under acidic conditions, suggesting its suitability for efficient sonodynamic therapy within the tumor microenvironment. Additionally, the piezoelectric field of CWO was confirmed under US irradiation through US current measurements (Figure 2c). Compared to the blank control group, CWO generated a strong acoustoelectric current signal under US irradiation, indicating superior piezoelectric properties when exposed to US conditions.

To evaluate the capacity of CWO to generate ROS, its VB, CB, and bandgap positions were extensively analyzed. The VB energy level of CWO was found to be ≈1.75 eV, determined by XPS (Figure 2d). Furthermore, the UV–vis absorption spectra were assessed, and the bandgap of CWO was calculated to be ≈1.67 eV using the Tauc plot derivative method (Figure 2e). Based on these results, the CB position was estimated to be 0.04 eV. Interestingly, the bandgap of CWO was found to be narrower compared to other representative piezoelectric sonosensitizers (Table S1, Supporting Information), which can facilitate •OH generation under US. Thereby, the mechanism for the US‐induced piezoelectric property of CWO is illustrated in Figure 2f. In the absence of US exposure, the intrinsic VB edge of CWO (+1.75 eV) is less positive than the redox potential of H_2_O/•OH (+2.10 V), making the hole energetically unfavorable for the redox reaction. Under US irradiation, changes in the local dipole moment of CWO lead to piezoelectric polarization, generating an inherent piezoelectric field. Specifically, this US‐induced mechanical deformation results in the generation of an internal electric field, promoting substantial band bending of the valence and conduction bands. Such band bending effectively enhances the spatial separation of electrons and holes, significantly reducing their recombination rate. As a result, the prolonged lifetime and improved availability of these charge carriers facilitate the direct oxidation of water molecules by holes, efficiently producing •OH.^[^ ^26^, ^30^, ^31^, ^32^, ^33^, ^34^ ^]^ Nevertheless, since the CB potential of CWO (+0.04 eV) is considerably more positive than the reduction potential required for •O_2_^−^ (−0.33 eV), it is theoretically unfavorable for CWO to produce •O_2_^−^ radicals under US irradiation.

After theoretical investigation, the capabilities of CWO to generate •OH and deplete GSH were subsequently explored (Figure 2g). The production of •OH radicals was first assessed using electron spin resonance (ESR) spectroscopy (Figure 2h). A characteristic 1:2:2:1 quartet ESR signal was detected, indicating the formation of •OH radicals in the CWO+US system, but this signal was not observed in the CWO‐only system (Figure S10, Supporting Information). This finding suggests that CWO can effectively generate •OH under US stimulation. Moreover, the production of •OH was promoted by H_2_O_2_, indicating that the US further boosts •OH production in H_2_O_2_‐rich environments, such as those typically found in tumor microenvironments. Additionally, the •OH production ability of CWO was further examined using 3,3’,5,5’‐tetramethylbenzidine (TMB) as an indicator (Figure 2i). The CWO+H_2_O_2_ group catalyzed the oxidation of TMB into its blue‐colored oxidized form (ox‐TMB). Notably, this effect was further amplified under US irradiation, providing additional evidence that the US enhances the •OH production capability of CWO. Furthermore, the •OH generation was positively related to the concentrations of CWO and period of US (Figure S11, Supporting Information). These findings collectively demonstrate the potential of CWO for enhanced •OH generation, particularly in H_2_O_2_‐rich environments under US irradiation. Furthermore, the capacity of CWO to generate ^1^O_2_ was systematically assessed using ESR spectroscopy and singlet oxygen sensor green (SOSG) fluorescence assays. Under continuous US irradiation, no characteristic ESR signals corresponding to ^1^O_2_ were observed. Consistently, the SOSG fluorescence intensity showed only a minimal increase relative to the control group. These results confirm that the sonodynamic effect of CWO does not depend on the generation of ^1^O_2_ (Figure S12, Supporting Information).

Subsequently, the ability of CWO to oxidize GSH to glutathione disulfide (GSSG) was examined using 2‐dithiobis(2‐nitrobenzoic acid) (DTNB). As DTNB can react with non‐oxidized GSH to form 2‐nitro‐5‐thiobenzoic acid (TNB), the absorbance of TNB was analyzed to quantify the GSH consumption. As shown in Figure 2j, when CWO was incubated with GSH, the characteristic absorbance of TNB gradually decreased over time, and this decrease was found to be positively related to the concentration of CWO. This indicates that CWO effectively and continuously consumed GSH. Moreover, the generated holes in the VB of CWO during US stimulation can further accelerate the depletion of GSH. Thereby, under US stimulation, the rate of GSH depletion was found to be significantly enhanced, indicating that US can amplify CWO's capacity to deplete GSH (Figure S13, Supporting Information).

Lastly, the release of Cu^2+^ from CWO was investigated by inductively coupled plasma (ICP). Figure 2k showed that under US irradiation, the release of Cu^2+^ from CWO was promoted from ≈1.2%, ≈2.6%, and ≈3.8% to ≈5.1%, ≈8.0%, and ≈10.0% at pH 7.4, 6.0, and 5.0, respectively. These results confirmed that Cu^2+^ can be effectively released from CWO under US and acidic conditions.

**Figure 3:**
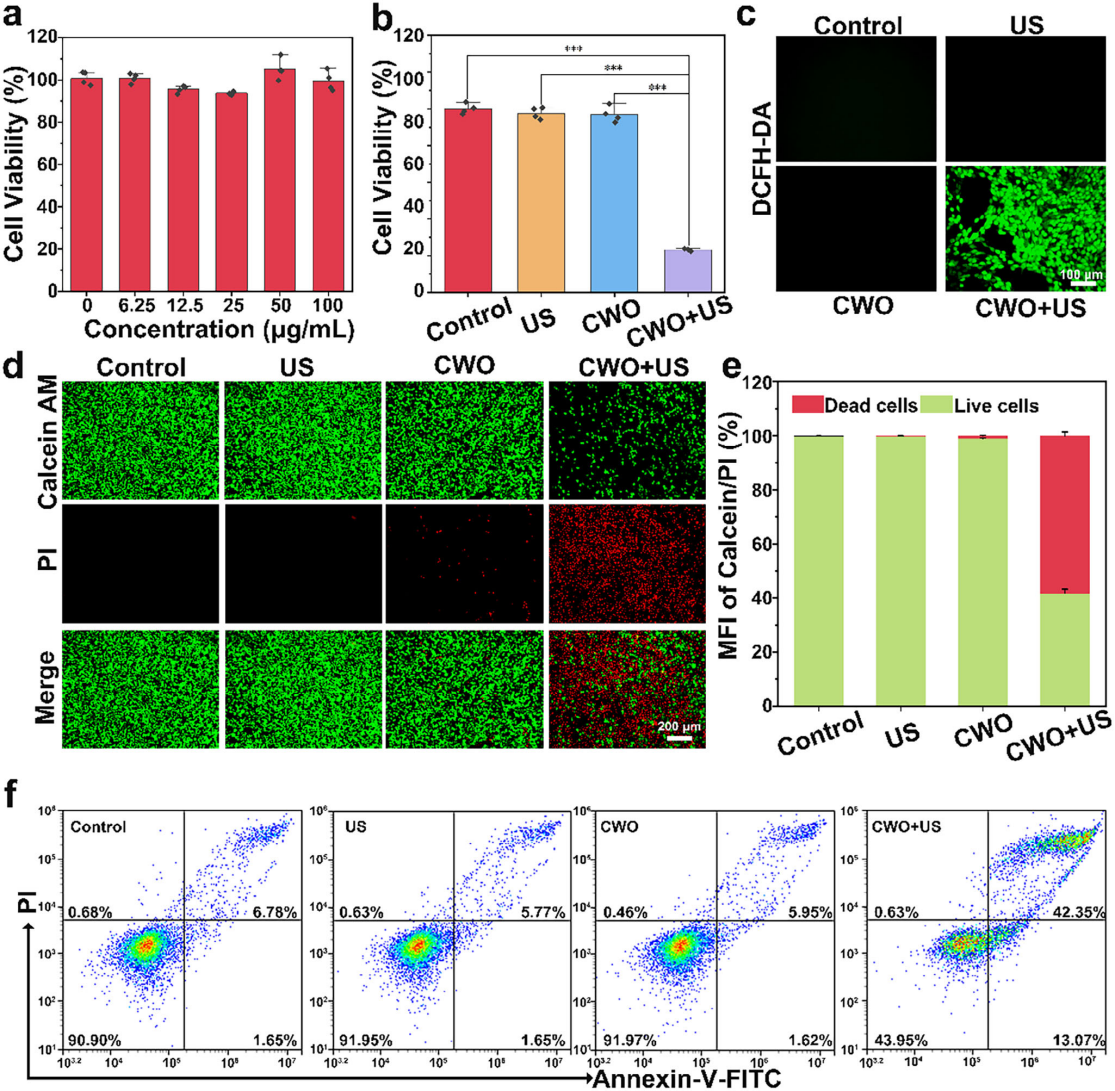
In vitro antitumor activity of CWO. a) CWO cytotoxicity in L929 cells. b) Therapeutic efficacy of CWO (100 µg mL^−1^) in Panc02 cells. c) DCFH‐DA staining and d) Calcein AM/PI staining after various treatments. e). Corresponding quantification of mean fluorescence intensity of Calcein AM/PI staining. f) Flow cytometry assay. Values are expressed as mean ± standard deviation (SD), (*n* = 3). Statistical analysis was performed by one‐way ANOVA. ^***^ *P* < 0.001.

Inspired by CWO's unique ability to generate substantial amounts of •OH under US, we proceeded to evaluate its in vitro therapeutic efficacy. To evaluate the cellular uptake of CWO, it was first labeled with Cy2 fluorescent dye and then incubated with Panc‐02 cells for different time periods (Figure S14, Supporting Information). CWO showed strong co‐localization with the cells, indicating efficient cellular uptake. In addition, the peak fluorescence signal was observed at 3h, therefore, US was applied at 3h for all subsequent in vitro experiments. Subsequently, the cytotoxicity of CWO was investigated using cell counting kit‐8 (CCK‐8) assays on L929 and Panc02 cell lines. The cell viability of CWO‐treated L929 and Panc02 cells remained above 80% when the concentration was ≈100 µg mL^−1^ after 24 h of co‐culture, indicating that CWO does not show substantial cytotoxic effects on these cells when US is not applied (**Figure** **3**a; Figure S15, Supporting Information). However, the inhibitory effect on panc02 cells was significantly enhanced with US irradiation. In the CWO+US treatment group, the viability of Panc02 cells drastically reduced to ≈20%, whereas other treatment groups maintained over 90% cell viability (Figure 3b). Moreover, the cytotoxic effect of different concentrations of CWO under US irradiation was further investigated via CCK‐8 assay, revealing that the therapeutic efficacy significantly increased in a concentration‐dependent manner (Figure S16, Supporting Information). These results demonstrate that CWO exhibits potent cytotoxicity toward Panc02 cells when activated by the US, highlighting the essential role of the US in unlocking the therapeutic potential of CWO against Panc02 cells. Subsequently, the intracellular generation of •OH was examined using the 2′,7′‐dichlorofluorescein diacetate (DCFH‐DA) probe (Figure 3c). Among the different groups, the CWO+US treatment exhibited intense green fluorescence, indicating a substantial •OH‐generating response when stimulated with the US (Figure S17, Supporting Information). Additionally, a live/dead cell assay was conducted, showing that the CWO+US induced the most cell death, whereas the CWO‐only group did not exhibit significant cell death. This is attributed to the significant •OH generation under US stimulation (Figure 3d,e). Furthermore, flow cytometry analysis confirmed that the CWO+US treatment led to significant cell death in Panc02 cells, displaying an apoptosis rate of 55.4%, compared to rates of 8.43%, 7.42%, and 7.57% observed in the control, US, and CWO groups, respectively (Figure 3f; Figure S18, Supporting Information).

**Figure 4:**
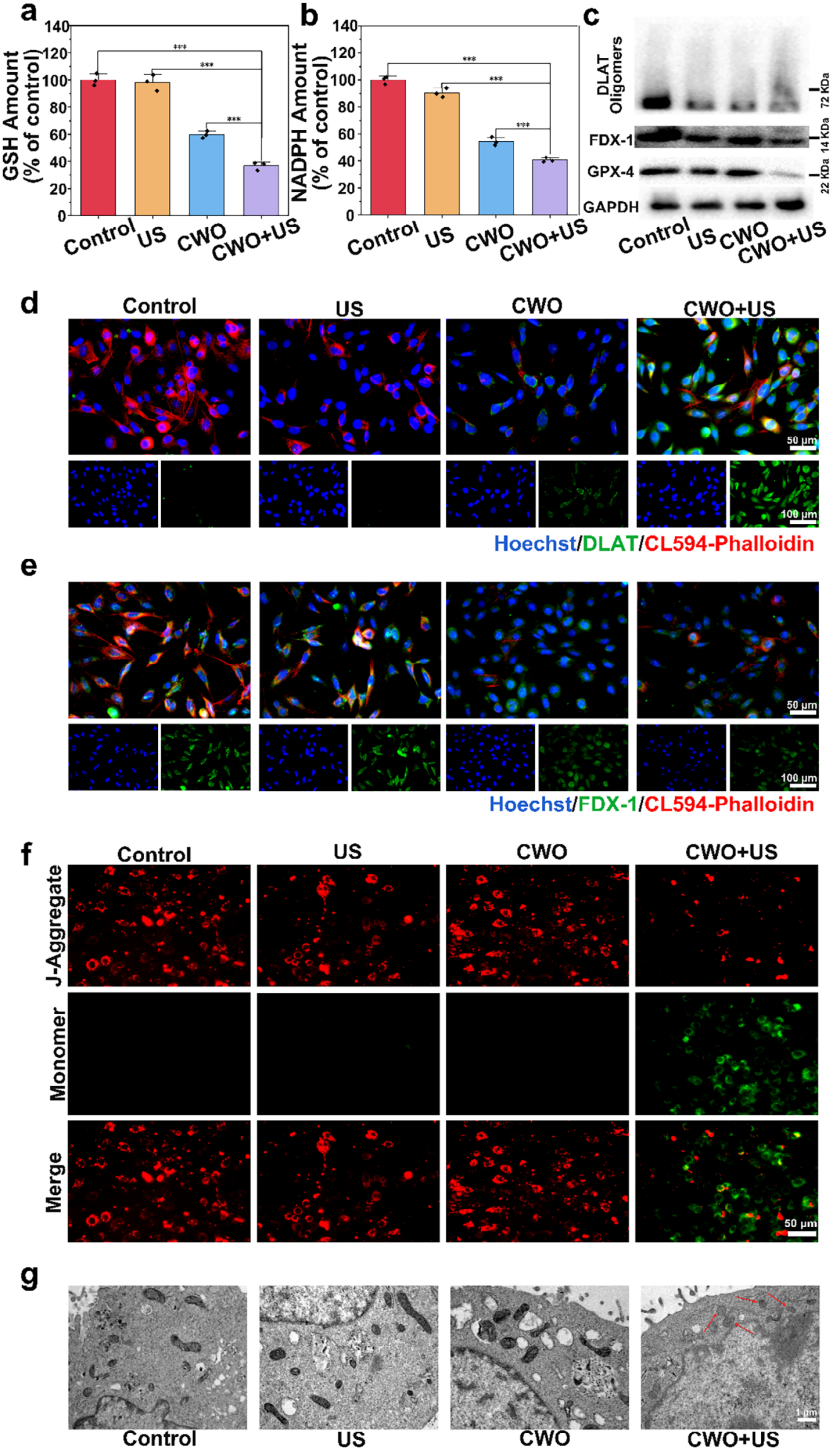
CWO‐mediated regulatory mechanisms of apoptosis signaling pathways in vitro. Quantitative evaluation of a) GSH and b) NADPH levels after different treatments. c) Western blot analysis on the expression of DLAT oligomers, FDX‐1, and GPX4. Fluorescence images of d) DLAT, e) FDX‐1, and f) JC‐1 probe after different treatments. g) Bio‐TEM visualization of mitochondrial morphology in Panc02 cells after various treatments. Values are expressed as mean ± SD (*n* = 3). Statistical analysis was performed by one‐way ANOVA. ^***^ *P* < 0.001.

The potent apoptotic effects induced by CWO+US treatment prompting a deeper investigation into the underlying mechanisms. Oxidative stress plays a pivotal role in antitumor therapy by disrupting redox homeostasis and inducing cellular damage. Besides inducing direct oxidative stress, depleting intracellular antioxidants, particularly GSH and NADPH, is essential for disrupting the redox balance. GSH serves as a primary cellular antioxidant, while NADPH is crucial for regenerating GSH and other antioxidants. The depletion of intracellular GSH and NADPH was shown in **Figure** **4**a,b. Compared to the control group, GSH levels were reduced to ≈65% in the CWO treatment group and further to 40% in the CWO+US group, indicating a synergistic effect of US in enhancing CWO‐mediated oxidative stress. Similarly, NADPH levels decreased significantly to ≈50% in the CWO group and to 35% with the CWO+US combined treatment. These results suggest that CWO+US can efficiently disrupt the redox homeostasis in tumor cells by depleting critical antioxidants, exacerbating oxidative damage. The consumption of GSH and NADPH is particularly significant in ferroptosis, as it leads to the reduced activity and expression of glutathione peroxidase 4 (GPX4).^[^ ^35^, ^36^ ^]^ As a result, a notably lower level of GPX4 expression was detected by Western blot (Figure 4c).

Building on the observed effects of CWO on intracellular GSH and NADPH depletion and subsequent GPX4 downregulation, we further explored CWO's ability to induce cuproptosis. For this purpose, two marker proteins in the relevant signaling pathways, DLAT and FDX‐1, were analyzed. DLAT is a component of the pyruvate dehydrogenase complex located in the mitochondria, and it plays a crucial role in regulating carbon entry into the TCA cycle. FDX‐1 is a Fe‐S cluster protein that acts upstream in the cuproptosis pathway. This reductase can convert Cu^2^⁺ to the more toxic Cu⁺ and regulate the lipoylation of DLAT, leading to its downregulation and thereby positively influencing the induction of cuproptosis.^[^ ^37^, ^38^ ^]^ Western blot analysis revealed that the total expression of DLAT protein was reduced following treatment with CWO+US (Figure 4c). This phenomenon was attributed to the binding of Cu ions to DLAT, which facilitates DLAT oligomerization and increases the insoluble fraction of DLAT, resulting in irreversible proteotoxic stress and cellular damage. Additionally, a significant downregulation of FDX‐1 expression was observed in the CWO+US group compared to the other groups. These findings collectively demonstrate that cuproptosis is effectively activated by CWO and is further amplified when combined with US irradiation.

Subsequently, the immunostaining of DLAT and FDX‐1 was conducted. Figure 4d showed the immunostaining of DLAT, a prominent green fluorescence was observed in the cytoplasm after CWO treatment, indicating significant aberrant DLAT oligomerization due to the released copper ions from CWO. The most intense DLAT fluorescence was seen in the CWO+US group, suggesting enhanced cuproptosis due to US stimulation (Figure S19, Supporting Information). In addition, as illustrated in Figure 4e, the FDX‐1 fluorescence was significantly reduced after CWO+US treatment (Figure S20, Supporting Information). The oxidative stress and cuproptosis triggered by CWO could lead to extensive mitochondrial oxidative damage, resulting in severe mitochondrial dysfunction.^[^ ^39^ ^]^ This mitochondrial impairment was assessed by measuring the mitochondrial membrane potential using JC‐1 (5,5',6,6'‐tetrachloro‐1,1',3,3'‐tetraethylbenzimidazolylcarbocyanine iodide) probe. In the CWO+US group, the monomer fluorescence signal was the strongest (Figure 4f; Figure S21, Supporting Information), which indicated a substantial reduction in mitochondrial membrane potential, reflecting marked mitochondrial dysfunction. To further investigate the extent of mitochondrial damage, biological TEM (bio‐TEM) was employed. The Bio‐TEM images revealed that mitochondria in cells treated with CWO+US were severely disrupted (Figure 4g), providing direct evidence of significant mitochondrial damage caused by this treatment. These findings conclusively demonstrated that CWO+US induced considerable mitochondrial dysfunction and damage.

**Figure 5:**
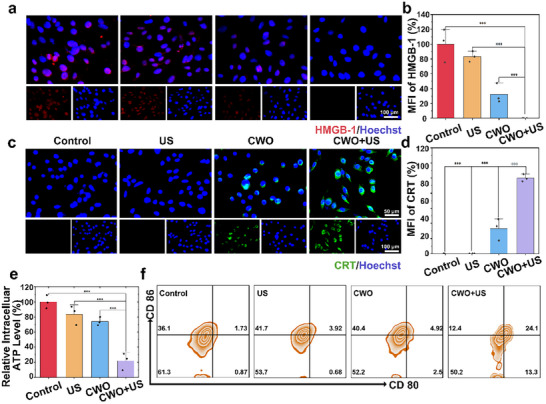
ICD induced by CWO. a) Fluorescence images of CRT after different treatments. b) Corresponding quantification of CRT (*n* = 3). c) Fluorescence images of HMGB‐1 after different treatments. d). Corresponding quantification of HMGB‐1 (*n* = 3). e) Intracellular ATP levels after different treatments. f) DCs maturation determined by flow cytometry. Values are expressed as mean ± SD (*n* = 3). Statistical analysis was performed by one‐way ANOVA. ^**^ *P* < 0.01 and ^***^ *P* < 0.001.

Mitochondrial damage can significantly disrupt mitochondrial integrity, leading to the release of damage‐associated molecular patterns (DAMPs) such as high‐mobility group box 1 (HMGB1), calreticulin (CRT), and adenosine triphosphate (ATP).^[^ ^8^, ^34^, ^40^ ^]^ Thereby, we further investigated the release of DAMPs after different treatments. First, we analyzed the release of HMGB1 from the cell nucleus. As shown in **Figure** **5**a,b, HMGB1 in the control group exhibited strong red fluorescence, predominantly localized in the nucleus. In contrast, the CWO+US group exhibited almost no red fluorescence, indicating significant HMGB1 release. Next, we evaluated the surface exposure of CRT. The CWO+US group exhibited the highest level of CRT exposure (Figure 5c,d). Furthermore, Western blot analysis of protein expression was performed, revealing a significant translocation of CRT to the cell membrane surface and a corresponding marked reduction of HMGB1 in the nuclear fraction in the CWO+US group compared to the control group, confirming CRT externalization and extracellular release of HMGB1 (Figure S22, Supporting Information). The dysfunction of mitochondria could cause efflux of intracellular ATP, thereby, the level of intracellular ATP in the CWO+US group was found to be markedly lower than in other groups, confirming that a large amount of ATP was released from the cell nucleus after the CWO+US treatment (Figure 5e). The release of DAMPs can lead to ICD, enhancing cancer immunotherapy.^[^ ^2^, ^36^, ^41^ ^]^ Therefore, to investigate this effect, we extracted bone marrow‐derived dendritic cells (BMDCs) from mice and co‐cultured them in vitro with supernatants from tumor cells subjected to various treatments. As shown in Figure 5f, the supernatant of the CWO+US group significantly enhanced dendritic cells (DCs) maturation, with maturation levels increasing from 1.73% in the control group to 24.1% (Figures S23 and S24, Supporting Information). These findings demonstrate that CWO, when activated by US, can efficiently damage mitochondria, release DAMPs, and trigger ICD, thereby enhancing immune responses and highlighting its potential significance in tumor immunotherapy.

After evaluating the in vitro therapeutic effect of CWO, the in vivo therapeutic analysis was then conducted. First and foremost, the biosafety of CWO is thoroughly evaluated. The hemolytic test was conducted with varying concentrations of CWO, which showed no signs of hemolytic activity (Figure S25, Supporting Information). Furthermore, the biosafety was assessed by injecting CWO and PBS into healthy mice via the tail vein. Hematological parameters showed no significant differences between the CWO‐treated group and the control group (Figure S26, Supporting Information). Additionally, the impact of CWO on kidney and liver function was minimal (Figure S27, Supporting Information), indicating that CWO does not compromise host biosafety. Tissue samples from major organs of mice in various treatment groups were collected and stained with hematoxylin and eosin (H&E). The staining results showed no significant morphological alterations in the organs of CWO‐treated mice compared to those treated with PBS (Figure S28, Supporting Information). Overall, these findings demonstrate that CWO is safe for in vivo application.

Subsequently, the in vivo biodistribution of CWO was investigated. Mice with Panc02 tumors were administered intravenously with Cy5.5‐labeled CWO (CWO@Cy5.5). At various time points, major organs and tumors were collected for fluorescence imaging. Ex vivo biodistribution analysis revealed that CWO began to accumulate in the tumor region within 1 h after injection and continued to increase. By 9 h post‐injection, the highest fluorescence intensity was observed in the tumor, thus, US was applied after 9 h of injection for all following in vivo investigations (Figures S28 and S30, Supporting Information).

**Figure 6:**
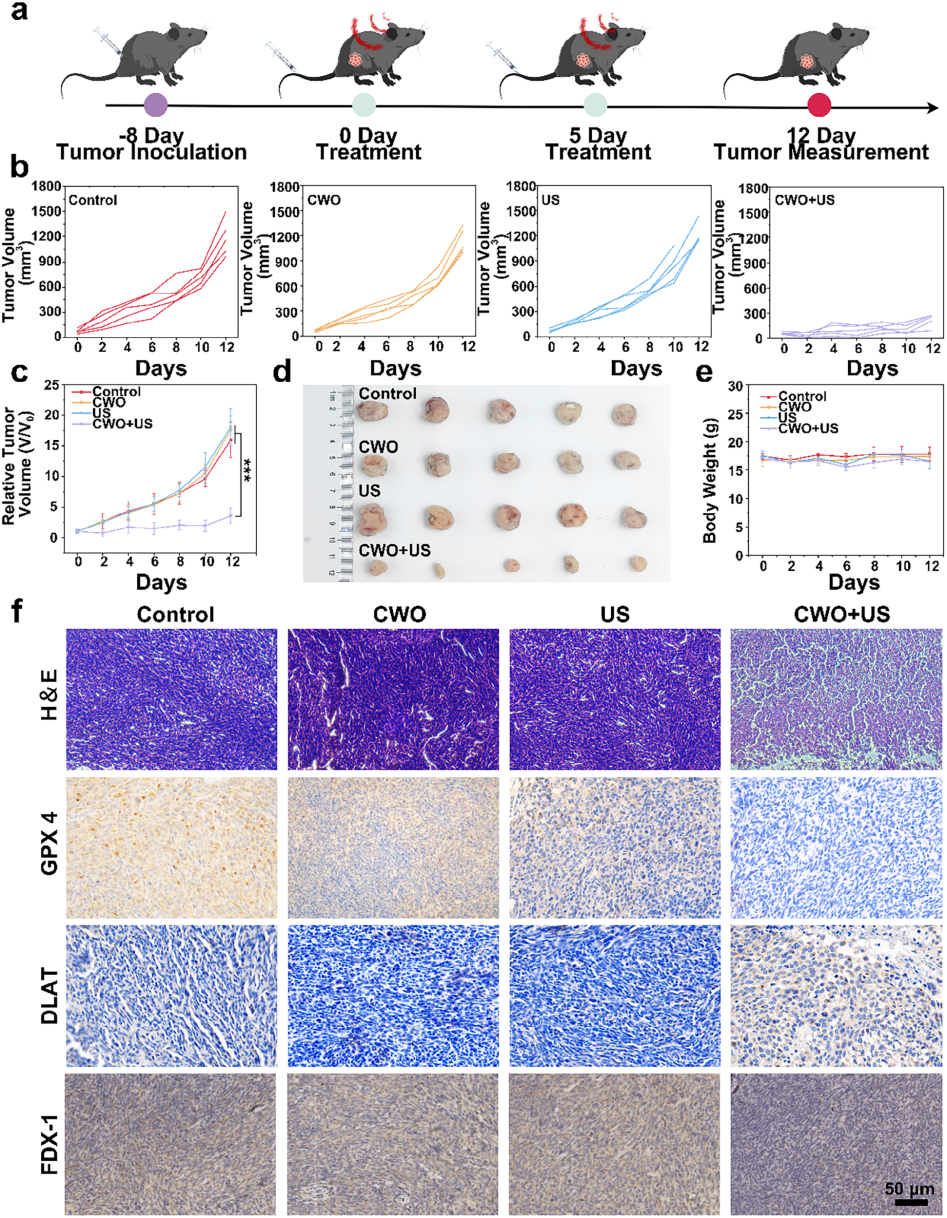
In vivo anti‐tumor effect of CWO. a) Schematic of the treatment. b) Tumor volume growth, c) Relative growth curves, d) Digital tumor photographs, and e) Body weight of mice after different treatments. f) H&E, GPX‐4, DLAT, and FDX‐1 staining after different treatments. Values are expressed as mean ± SD (*n* = 3). Statistical analysis was performed by one‐way ANOVA. ^***^ *P* < 0.001.

Next, the potential antitumor effects of CWO were evaluated in a Panc02 mouse model. Male C57BL/6 mice were inoculated subcutaneously with Panc02 tumor cells. When the tumor volume reached ≈60 mm^3^, the mice were randomly divided into four groups: PBS, CWO, US, and CWO+US. PBS and CWO were administered via intravenous injection on 0 and 5 days, with the US performed 9 h after injection. The therapeutic process was monitored over 12 days (**Figure** **6**a). Tumor sizes were recorded and summarized (Figure 6b), and the group receiving the combined CWO+US treatment showed significantly enhanced tumor inhibition (Figure 6c,d). These results indicate that the combination of CWO+US delivers the strongest antitumor effect. Additionally, the no significant changes in body weights of the mice throughout the different treatment groups, suggesting that the treatments did not cause adverse effects on overall health (Figure 6e).

To further assess the therapeutic efficacy, H&E staining and terminal deoxynucleotidyl transferase‐mediated dUTP‐biotin nick end labeling (TUNEL) staining were performed to evaluate tumor cell morphology and apoptosis. Microscopic examination of H&E and TUNEL‐stained tumor sections revealed that the CWO+US group showed the most extensive cellular damage and apoptosis (Figure 6f; Figure S31, Supporting Information). Additionally, immunohistochemical analysis of Ki‐67 revealed that the CWO+US group had the most pronounced decrease in cell proliferation, suggesting a significant reduction in tumor aggressiveness (Figure S32, Supporting Information). The expression of GPX4 was found to be significantly reduced in the CWO+US group, suggesting substantial ferroptosis induced by the combined treatment. Furthermore, consistent with the in vitro results, a marked decrease in the expression of key cuproptosis‐related proteins, FDX1 and DLAT, was observed in the tumor tissues treated with CWO+US (Figure 6f). Collectively, these findings indicate that CWO+US could effectively induce ferroptosis and cuproptosis, thereby inhibiting tumor growth in vivo.

**Figure 7:**
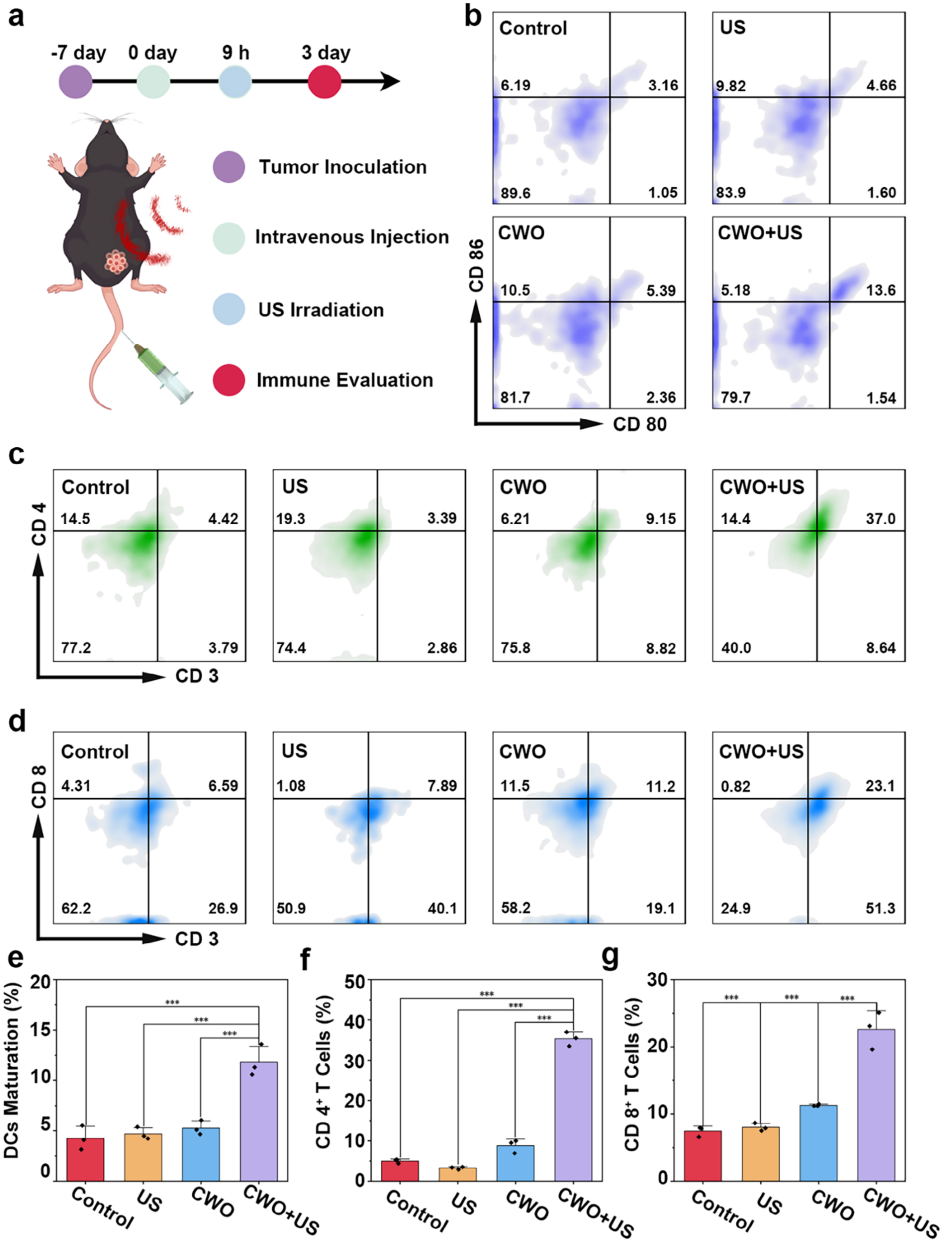
In vivo immune activation from CWO. a) Schematic of the treatment. b) Flow cytometry of DCs maturation in the lymph nodes after treatments. c) Flow cytometry of CD4^+^ T cell infiltration in the tumor. d) Flow cytometry of CD8^+^ T cell infiltration in the tumor tissue. e) Quantitative evaluation of DCs maturation. f) Quantitative evaluation of CD4^+^ T cells. g) Quantitative evaluation of CD8^+^ T cells. Values are expressed as mean ± SD (*n* = 3). Statistical analysis was performed by one‐way ANOVA. ^***^ *P* < 0.001.

Building upon the promising in vivo therapeutic effects, we then investigate the ICD induced by the cascade immunotherapy of CWO. First, the maturation of DCs were examined by analyzing the expression levels of CD80 and CD86 (**Figure** **7**a) While both CWO and US alone elicited moderate increases in DCs maturation compared to the control group, the CWO+US treatment significantly enhanced the expression of CD80 and CD86, the DCs maturation ratio was increased from 3.16% to 13.6% (Figure 7b,e). This significant enhancement in DCs maturation observed with CWO+US treatment underscores its capacity to initiate a robust immune response by promoting antigen presentation. Mature DCs play a pivotal role in bridging innate and adaptive immunity by migrating to lymph nodes and presenting tumor‐associated antigens to naïve T cells. Building on these findings, the subsequent analysis focuses on T‐cell activation, particularly the expansion of CD4^+^ helper T cells and CD8^+^ cytotoxic T cells. Figure 7c depicts the activation of CD4^+^ helper T cells, a critical component of adaptive immunity. The CWO+US treatment markedly increased the population of CD4^+^ T cells (37.0%) compared to the control group and individual treatments (4.42%, 3.39%, 9.15% in the control, CWO, and US groups, respectively) (Figure 7f). Similarly, the activation of cytotoxic CD8^+^ T cells, essential for tumor eradication, is shown in Figure 7d. While minimal activation was observed in the control and single‐treatment groups (6.59%, 7.89%, 11.2% in the control, CWO, and US groups, respectively), the CWO+US treatment significantly increased the activation of CD8^+^ T cells (23.1%) (Figure 7g). These results indicate that the combination therapy effectively bridges innate and adaptive immunity, driving a strong cytotoxic response against tumor cells.

**Figure 8:**
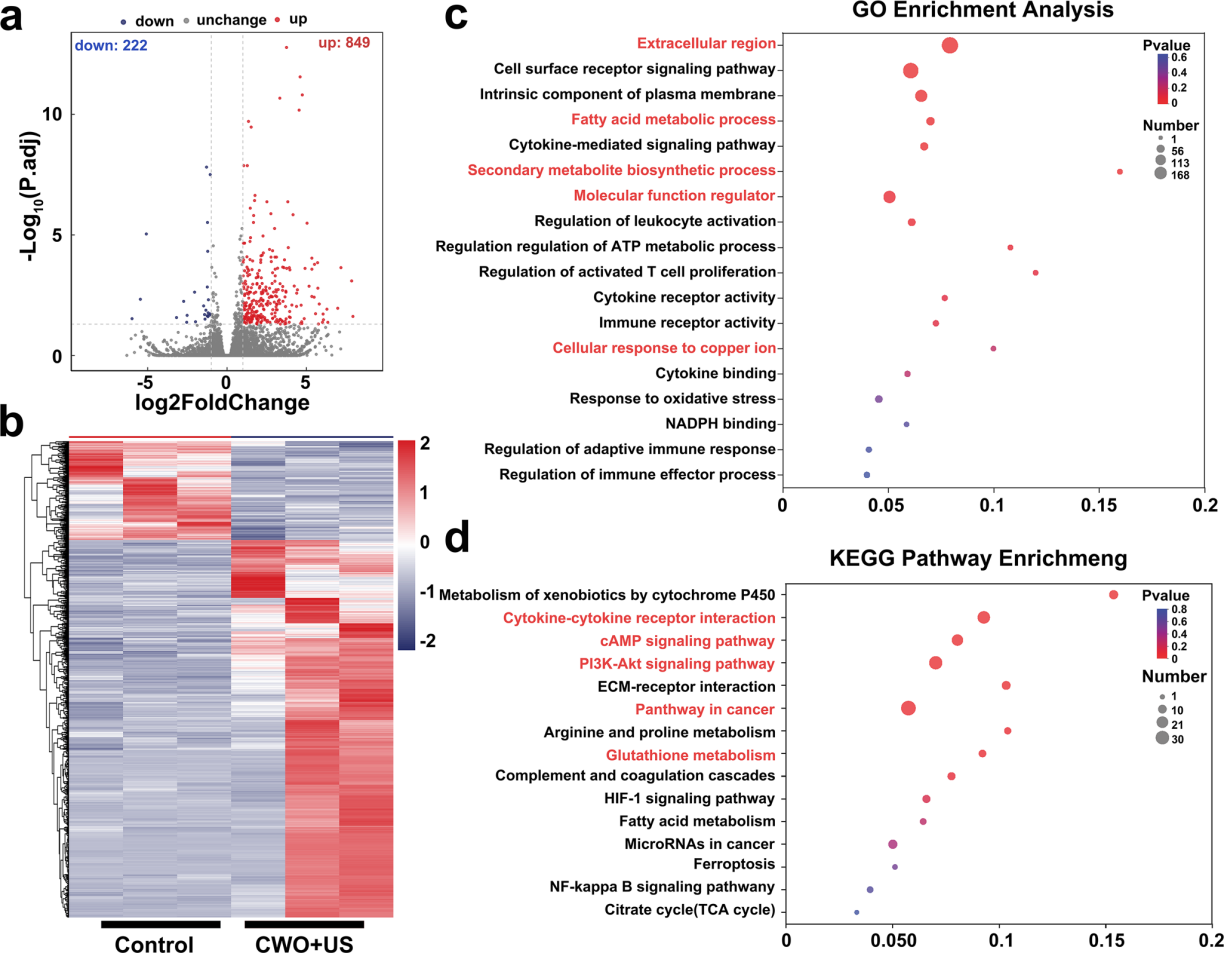
Transcriptomic analysis of tumor tissues after CWO+US treatment. a) Volcano plot, b) Heatmap comparing the control and CWO+US group. c) Gene Ontology (GO) enrichment analysis and Kyoto Encyclopedia of Genes and Genomes (KEGG) pathway enrichment analysis of genes elevated in the CWO+US group.

To further elucidate the underlying molecular mechanisms associated with CWO‐induced cuproptosis and immune activation, RNA‐seq analysis was performed on tumor tissues from the control and CWO+US groups post‐treatment. Differential gene expression analysis revealed substantial changes between these two groups, with a total of 849 upregulated and 222 downregulated genes (**Figure** **8**a). Hierarchical clustering illustrated by the heatmap clearly differentiated these gene expression patterns, further substantiating distinct transcriptional alterations upon CWO+US treatment (Figure 8b). To gain deeper insights into the biological relevance of these changes, Gene Ontology (GO) and Kyoto Encyclopedia of Genes and Genomes (KEGG) enrichment analyses were performed. GO enrichment analysis identified significantly enriched biological processes such as metabolic processes involving glucose, NADP, and glutathione; biosynthetic processes including ATP and NADPH synthesis; cellular responses to copper ions; inflammatory cytokine signaling; and adaptive immune responses (Figure 8c). These findings suggest that the cytotoxic mechanism of CWO involves intensified oxidative stress, disruption of cellular metabolism and biosynthesis, disturbance of copper ion homeostasis, and consequently the induction of ferroptosis and cuproptosis, as well as immune activation. KEGG pathway enrichment analysis further confirmed significant perturbations in multiple critical signaling pathways, including inhibition of glutathione metabolism, alterations in the PI3K‐Akt signaling pathway, TCA cycle disruptions, cAMP signaling, cytokine‐cytokine receptor interactions, chemokine signaling pathways, and several other immune‐related pathways closely associated with copper toxicity and immune modulation (Figure 8d). These data demonstrate that CWO‐mediated cascade therapy effectively suppresses tumor growth and enhances antitumor immunity through multifaceted molecular pathways in vivo.

In summary, CWO+US induces mitochondrial damage, releasing large quantities of cellular contents and DAMPs. These DAMPs promote the maturation of DCs, which subsequently migrate to lymph nodes and present tumor antigens to T cells. The activated T cells, both CD4^+^ and CD8^+^, then infiltrate the tumor microenvironment, eliciting a potent antitumor immune response. RNA‐seq analyses further support these observations by revealing significant transcriptional changes after CWO+US treatment, including enhanced oxidative stress, altered cellular metabolism, disruption of copper ion homeostasis, activation of ferroptosis and cuproptosis pathways, and modulation of multiple immune‐related signaling pathways. These molecular insights confirm the underlying mechanisms by which CWO+US effectively integrates piezoelectric catalytic activity, ferroptosis, cuproptosis, and immune cascade activation. Collectively, these results underscore the promise of CWO+US as an innovative therapeutic approach that harnesses piezoelectric sonosensitizers, ferroptosis, cuproptosis, and ICD. This synergistic strategy paves the way for effective and precise antitumor interventions.

## 3 Conclusion

In conclusion, CWO nanoparticles were successfully synthesized using a one‐pot hydrothermal method. When US is applied, CWO generates a significant amount of •OH via a piezoelectric‐catalysis mechanism, directly causing oxidative stress in cancer cells. Furthermore, CWO depletes GSH and NADPH, which exacerbates oxidative stress and initiates ferroptosis. More importantly, the release of Cu ions from CWO under acidic tumor conditions and the influence of US leads to cuproptosis in tumor cells. The combined effects of enhanced oxidative stress, ferroptosis, and cuproptosis ultimately result in mitochondrial dysfunction and the release of DAMPs, thereby inducing ICD and boosting cancer immunotherapy. This triple‐action strategy exhibited robust antitumor efficacy both in vitro and in vivo. Overall, our findings suggest a promising approach for precise tumor cell elimination through a US‐enhanced cuproptosis‐based therapy, providing a novel and potent method for cancer treatment.

## 4 Experimental Section

### Materials

Na_2_WO_4_, CuCl_2_, GSH, and DTNB were purchased from Aladdin Ltd. DLAT and FDX‐1 antibody were obtained from Sanying Ltd. Panc02 and L929 cells were acquired from the American Type Culture Collection.

### Synthesis of CWO

Briefly, CuCl_2_ (42.6 mg mL^−1^, 10 mL) was mixed with Na_2_WO_4_ (82.5 mg mL^−1^, 10 mL). Sodium citrate solution (60 µg mL^−1^ in 5 mL) was added, and the mixture was transferred into the autoclave tank and heated at 180 °C for 12 h. After washing and dialyzed with water for 24 h, CWO was obtained.

### Characterization

TEM images were captured using JEM‐2100. XRD spectra were obtained by the DMAX‐2400 X‐ray diffraction instrument. The ESCALAB 250 instrument was used for XPS. A UV‐1901 spectrophotometer was used for UV–vis spectra. ESR spectra were obtained using the Bruker EMX1598 spectrometer.

### In Vitro Detection of •OH Production

Release of •OH was measured using TMB. In a typical process, TMB (1 mg mL^−1^, 0.15 mL), H_2_O_2_ (1 mm, 300 µL), and CWO (1.2 mg mL^−1^, 250 µL) were mixed in DI water (2.3 mL). The US was applied for 3 min (1 W cm^−2^, 100% duty cycle, 1 MHz), and the absorbance was recorded by UV–vis.

### In Vitro Detection of ^1^O_2_ Production

Release of ^1^O_2_ was measured using SOSG. In a typical process, SOSG (1 mg mL^−1^, 0.15 mL) and CWO (1.2 mg mL^−1^, 250 µL) were mixed in DI water (2.3 mL). The US was applied for 3 min (1 W cm^−2^, 100% duty cycle, 1 MHz), and the absorbance was recorded by FL.

### ESR Measurement of •OH

10 µL CWO (10 mg mL^−1^), 10 µL H_2_O_2_ (1 m), DI water (70 µL) was mixed with 10 µL DMPO. Then •OH with the characteristic 1:2:2:1 signal was measured by ESR (Monitoring range: 3290–3370 G; Sweep time: 60 s; Freq.: 100 kHz).

### ESR Measurement of ^1^O_2_

10 µL CWO (10 mg mL^−1^), DI water (80 µL) was mixed with 10 µL TEMP 500 mm. Then ^1^O_2_ with the characteristic 1:1:1 signal was measured by ESR (Monitoring range: 320–360 G; Sweep time: 60 s; Freq.: 100 kHz).

### GSH Consumption

GSH (100 µm) and CWO (25 µg mL^−1^) were mixed, then DTNB was added. At a different time, the characteristic of TNB was detected by UV–vis.

### Cu^2+^ Release

CWO was dispersed in 1 mL of PBS at different pH levels in dialysis bags, which were placed into 10 mL of the respective pH buffered solutions. Cu^2+^ release was measured by ICP‐OES.

### Cell Culture

Panc02 and L929 cells were maintained in DMEM medium containing 10% fetal bovine serum and 1% penicillin/streptomycin, under humidified conditions at 37 °C with 5% CO_2_.

### Cellular Endocytosis Study

Panc02 cells were cultured for 24 h. Subsequently, Cy2‐labeled CWO was added.

### In Vitro Cytotoxicity

Panc02 cells were cultured for 24 h. Different concentration of CWO in 100 µL culture media was added and co‐incubated for 24 h. CCK‐8 (100 µL) was then added and incubated for another 1 h before analysis.

### Cell Viability After Treatments

CWO at different concentrations was co‐cultured with cells for 24 h. After 3h incubation, US was applied (1 W cm^−2^, 50% duty cycle, 3 min), and co‐cultured for an additional 18 h before adding CCK‐8 solution.

### Detection of Intracellular ROS Production

After co‐incubating with CWO for 3 h, DCFH‐DA was added and US was applied (1 W cm^−2^, 50% duty cycle, 3 min). Co‐incubating for 20 min before fluorescence imaging.

### Live/Dead Assay

As in the previous experiment, cells were treated with CWO (100 µg mL^−1^) with various treatments. Then, the cells were incubated for another 18 h before staining with Calcein‐AM and PI. Co‐incubating for 20 min before fluorescence imaging.

### Apoptosis Detection Assay

Similar to the previous experiment, after treatment, cells were detached by trypsin, and Annexin V‐FITC/PI was added for flow cytometry.

### DLAT Oligomerization

As in the previous experiment, cells were treated with CWO (100 µg mL^−1^) with various treatments. Subsequently, the cells were fixed with 4% paraformaldehyde, incubated overnight with the DLAT antibody, followed by treatment with an Alexa Fluor 488 anti‐mouse secondary antibody for 1 h. They were then stained with Actin‐Tracker Red for 30 min and Hoechst 33342 for 10 min, after which immunofluorescence imaging was performed.

### Loss of Fe–S Cluster Proteins FDX‐1

Identical to DLAT immunofluorescence imaging procedures, the FDX‐1 antibody was used for FDX‐1 immunofluorescence imaging.

### Mitochondrial Membrane Potential Detection

As in the previous experiment, cells were treated with CWO (100 µg mL^−1^) with various treatments. After an additional 4 h incubation, the cells were stained with JC‐1 according to the manufacturer's standard protocol.

### Evaluation of Mitochondrial Morphology

As in the previous experiment, cells were treated with CWO (100 µg mL^−1^) with various treatments. To analyze mitochondrial morphology, the treated cells were collected, fixed, and prepared as ultrathin sections for Bio‐TEM observation.

### Immunofluorescence Analysis of CRT Expression

As in the previous experiment, cells were treated with CWO (100 µg mL^−1^) with various treatments. After the treatments, the cells were fixed with 4% paraformaldehyde and stained with CRT antibody for immunofluorescence imaging.

### Immunofluorescence Analysis of HMGB‐1 Expression

Identical to CRT immunofluorescence imaging procedures, the HMGB‐1 antibody was used for HMGB‐1 immunofluorescence imaging.

### ATP Assays

As in the previous experiment, cells were treated with CWO (100 µg mL^−1^) with various treatments. The intracellular ATP concentrations were identified with the ATP Assay Kit.

### NADPH Assays

As in the previous experiment, cells were treated with CWO (100 µg mL^−1^) with various treatments. The cells were then lysed using a cell lysis solution. The supernatant was collected and incubated with chromogenic solution at 37 °C for 1 h, and the absorbance was measured at 450 nm.

### Detection of GSH Depletion at the Cellular Level

As in the previous experiment, cells were treated with CWO (100 µg mL^−1^) with various treatments. Then, co‐culture for another 3 h. The cells were then lysed using a cell lysis solution. The supernatant was collected and incubated with DTNB at 37 °C for 1 h, and the absorbance was measured at 412 nm.

### DCs Maturation Assay

Panc02 cells were seeded in 24‐well plates and cultured overnight. After removing the medium, cells were incubated with CWO (100 µg mL^−1^) for 3 h. As in the previous experiment, various treatments were applied under the same conditions, and the cells were cultured overnight. The supernatant was then collected. Meanwhile, the differentiated DCs were harvested and seeded in 24‐well plates. The collected supernatant from treated Panc02 cells was added to the cultures. After 24 h of co‐culture, the DCs were harvested and prepared as a single‐cell suspension. The cells were stained with antibodies: CD45‐FITC, CD11c‐Violet 450, CD80‐APC, and CD86‐PE‐Cy7. Finally, the cells were washed three times with PBS and analyzed by flow cytometry.

### Animal Experiences

All the procedures were approved by Shanghai University of Engineering Science (Approval No. EST‐2024‐013).

### Hemolysis of CWO

For the hemolysis assay, red blood cells (RBCs) were isolated from mouse blood by centrifugation at 1500 rpm for 5 min, washed with PBS, and diluted to a 10% suspension. The RBCs were then incubated with various concentrations of CWO (6.25, 12.5, 25, 50, 100 µg mL^−1^) for 1 h. Hemolysis rates were quantified by measuring the absorbance of the mixtures at 570 nm.

### Biocompatibility

Healthy C57BL/6 mice were administered CWO (15 mg kg^−1^) via intravenous injection. Blood samples were collected from the mice at 3‐, 7‐, and 30‐days post‐injection for routine hematological analysis.

### Tumor Suppression Experiments In Vivo

Mice received two intravenous injections (100 µL, 15 mg kg^−1^) of CWO. US treatment (2.0 W cm^−^^2^, 50% cycle, 5 min) was administered 9 h after the injection. Throughout the treatment period, tumor volume and body weight were monitored every two days. On day 12, the tumors were harvested and stained with H&E, TUNEL, Ki‐67, GPX‐4, DLAT, and FDX‐1 antibodies.

### Statistical Analysis

Quantitative data were expressed as the mean ± SD. Statistical comparisons were conducted using one‐way ANOVA. ^**^ *P* < 0.01 and ^***^ *P* < 0.001.

## Conflict of Interest

The authors declare no conflict of interest.

## Supporting information



Supporting Information

## Data Availability

The data that support the findings of this study are openly available in Advanced Science at https://doi.org/10.1002/advs.202500576.
