# Supplementary material for: High‐Efficiency Carriers’ Separation Strategy Based Ultrasmall‐Bandgap CuWO4 Sono‐Enhances GSH Antagonism for Cuproptosis Cascade Immunotherapy
Source: Adv Sci (Weinh). 2025 May 21;12(29):e00576. doi: 10.1002/advs.202500576 (PMC12362811; doi:10.1002/advs.202500576)
Supplement: Supplementary file 1 — Supporting Information [file ADVS-12-e00576-s001.docx]

**High-efficiency Carriers’ Separation Strategy Based Ultrasmall-Bandgap CuWO_4_ Sono-enhances GSH Antagonism for Cuproptosis Cascade Immunotherapy**

*Lichao Zhu, Zhisheng Guo, Yu Luo*, Haiyan Huang, Kexin Zhang, Bingbing Duan, Renmiao Peng, Haochen Yao*, Chao Liang*, Kaiyang Wang**

L. Zhu, Y. Luo, H. Huang, K. Zhang, B. Duan, R. Peng, K. Wang

*Shanghai Engineering Research Center of Pharmaceutical Intelligent Equipment, Shanghai Frontiers Science Research Center for Druggability of Cardiovascular Non-coding RNA, Institute for Frontier Medical Technology, School of Chemistry and Chemical Engineering Shanghai University of Engineering Science, Shanghai 201620, P. R. China*

Email: *yuluo@sues.edu.cn (Y. Luo);* [*kaiyang.wang@sues.edu.cn*](mailto:kaiyang.wang@sues.edu.cn) *(K. Wang)*

Z. Guo, C. Liang

*Department of Urology, The First Affiliated Hospital of Nanjing Medical University and Jiangsu Province Hospital, Nanjing 210029, P. R. China.*

*Email:* [*cliang@njmu.edu.cn*](mailto:cliang@njmu.edu.cn) *(C. Liang)*

H. Yao

*Hepatobiliary and Pancreatic Surgery Department, General Surgery Center, First Hospital of Jilin University, No.1 Xinmin Street, Changchun, 130021, Jilin, P. R. China.*

*Email: yaohaochen@jlu.edu.cn*


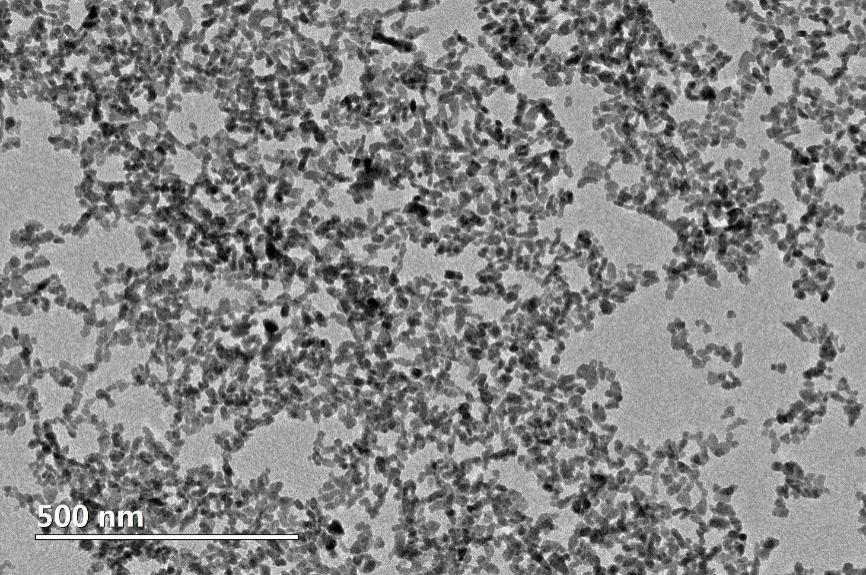


Figure S1 TEM image of CWO.


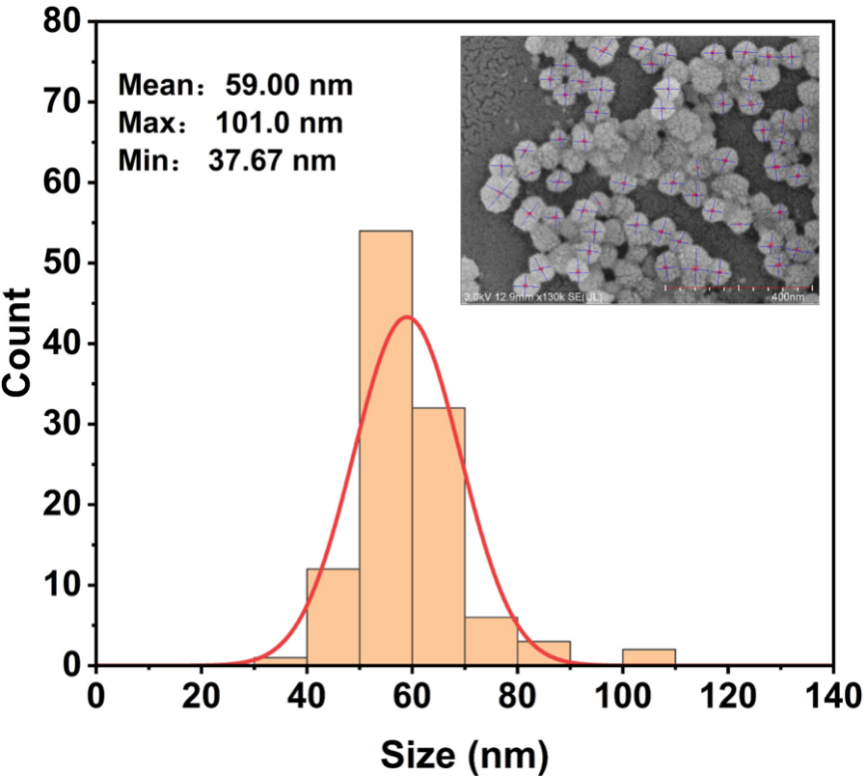


Figure S2 Particle size statistics of CWO in the SEM image.


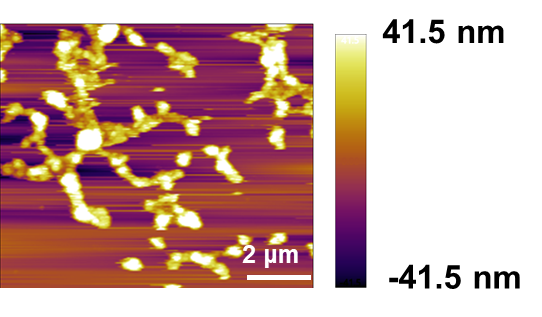


Figure S3 AFM image of CWO.


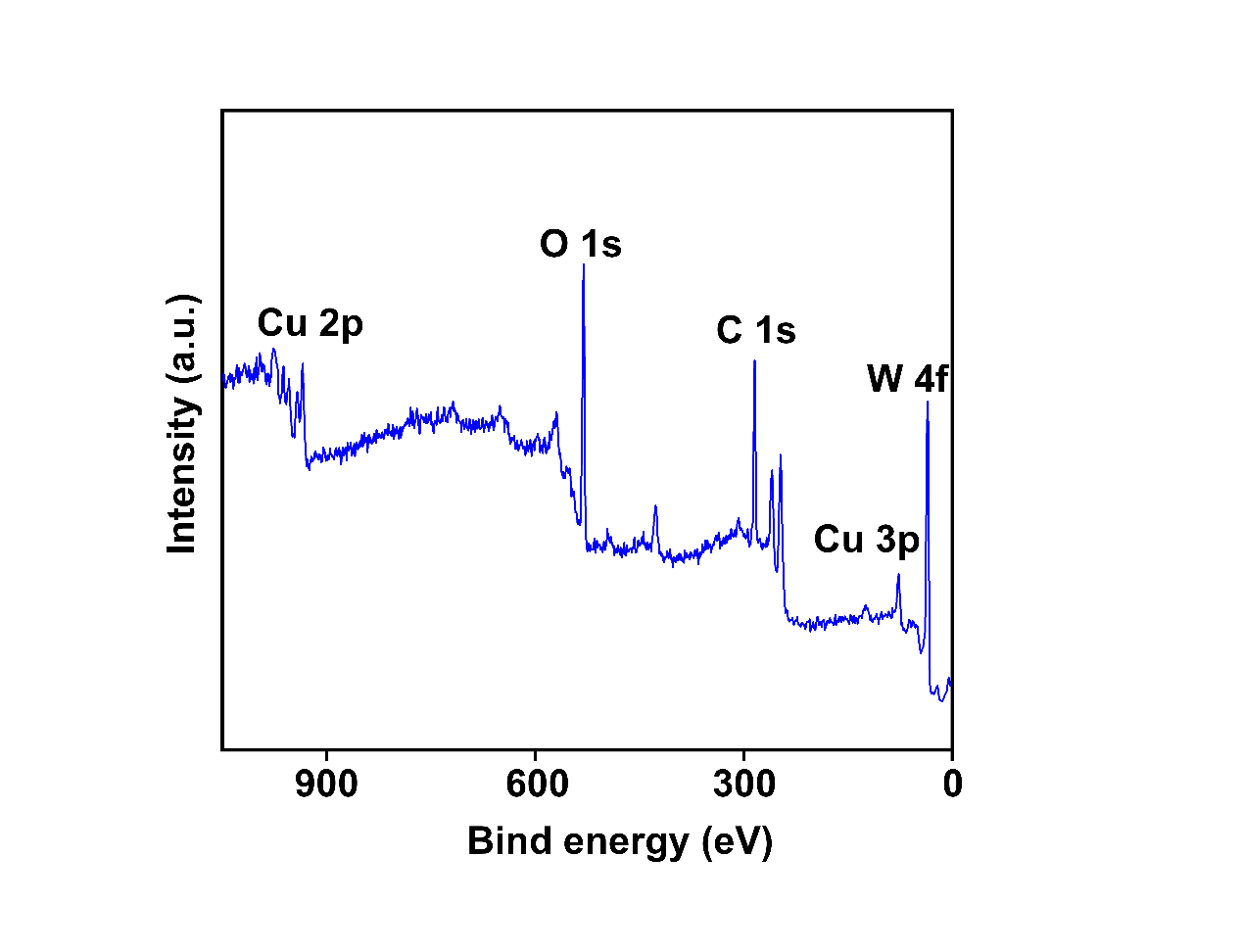


Figure S4 XPS spectra of wide-scan of the CWO.


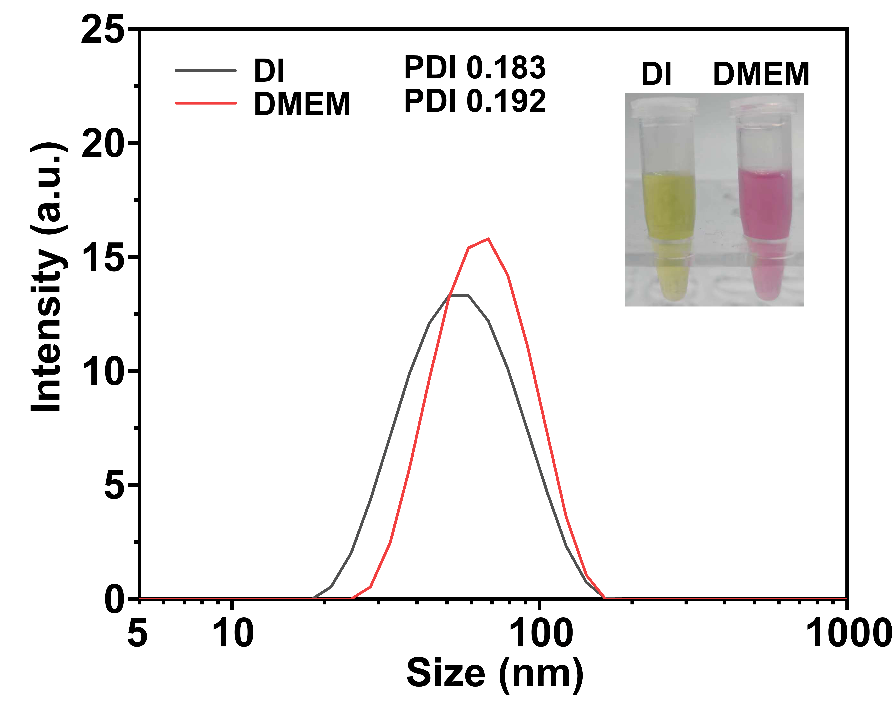


Figure S5 HD of the CWO dispersed solutions by DLS measurements.


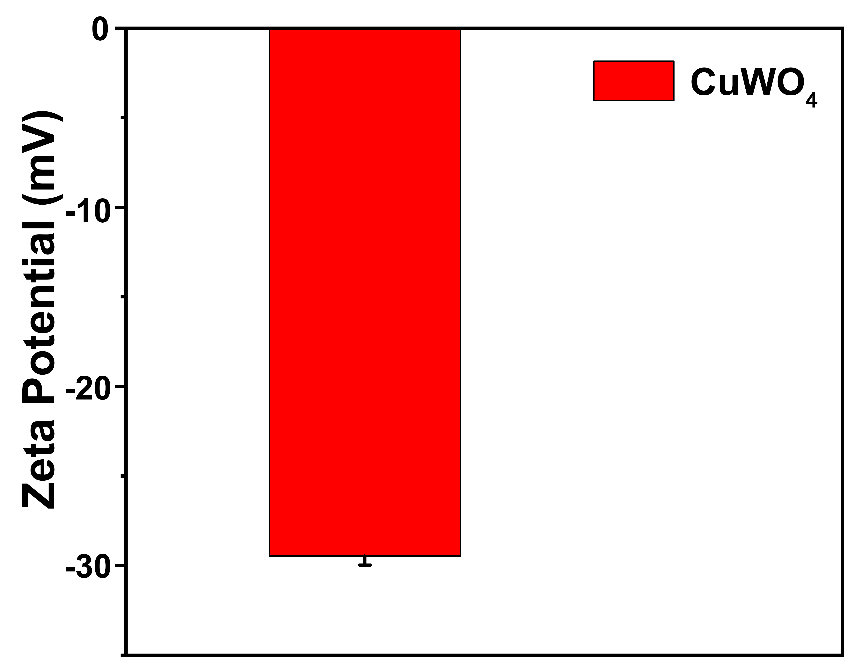


Figure S6 Zeta potential of CWO (n = 3, mean ± SD).


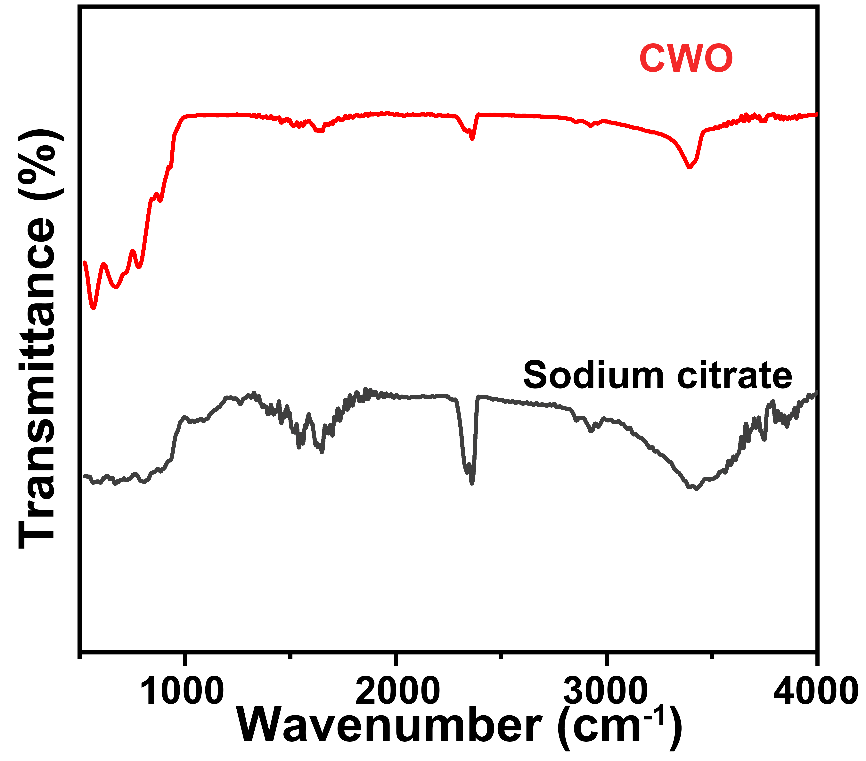


Figure S7 FTIR of CWO and Sodium citrate.


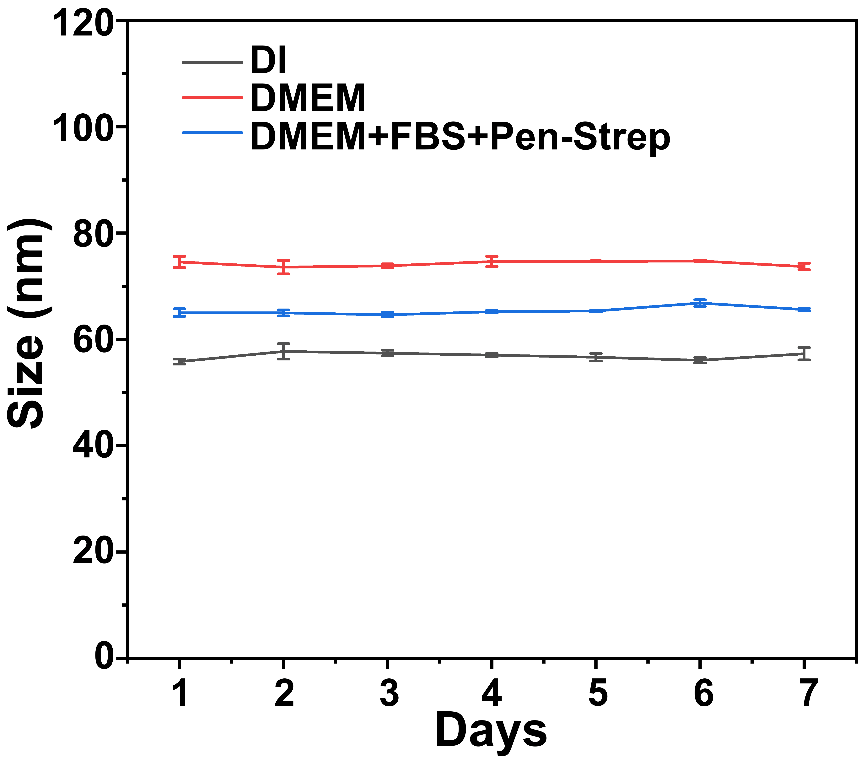


Figure S8 Hydrodynamic diameter (HD) of CWO after incubation in H_2_O、DMEM and DMEM + FBS + Pen-Strep for 7 days (n = 3, mean ± SD).


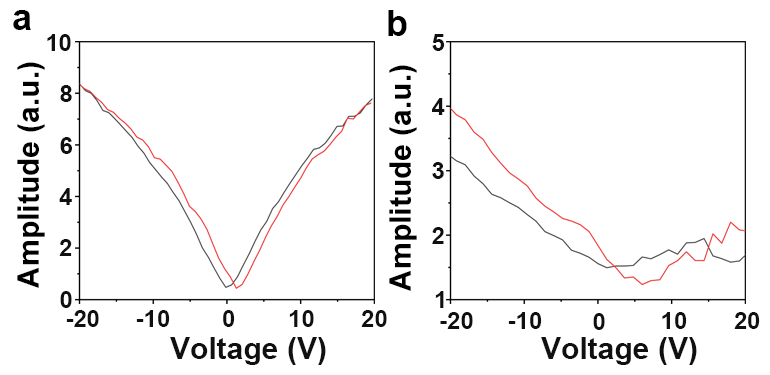


Figure S9 a) Butterfly curve of CWO (pH=5.0) using PFM. b) Butterfly curve of CWO (pH=9.0) using PFM.


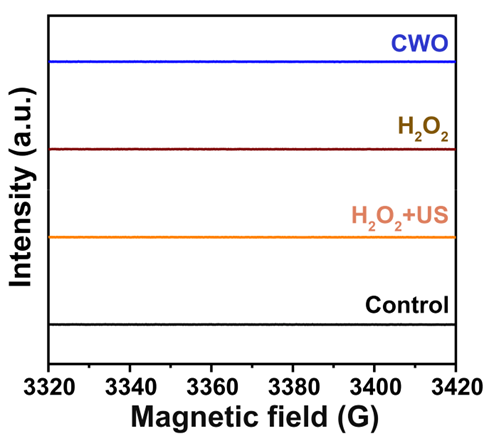


Figure S10 ESR spectra of CWO after various treatments.

Figure S11 a) Comparison of TMB oxidation after different treatments, US irradiation for 3 min. b) Absorption changes of TMB under different concentration of CWO. c) Time-dependent oxidation of TMB by CWO after different US time.


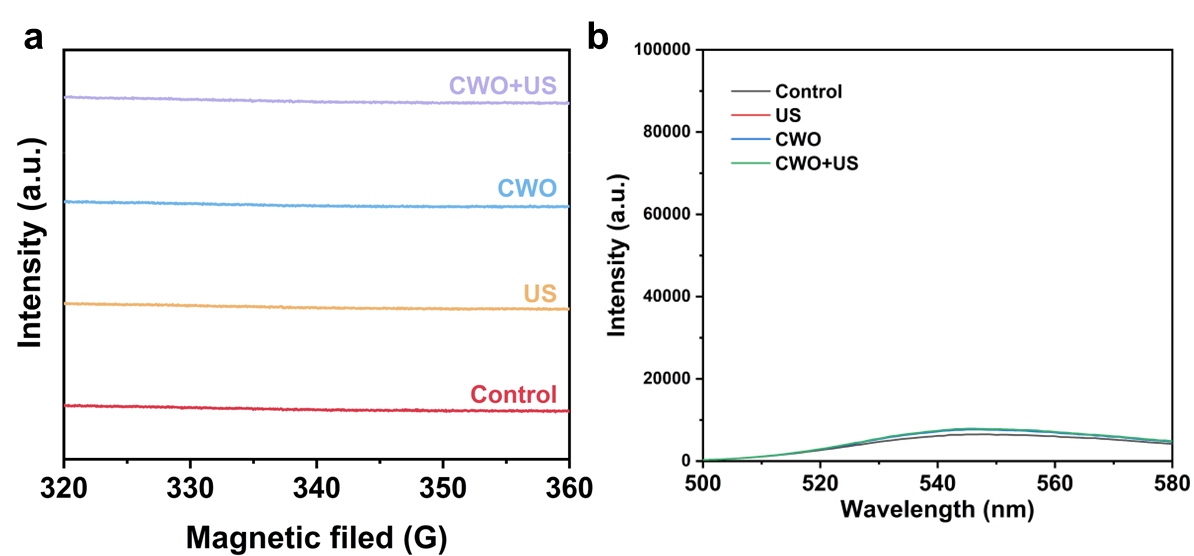


Figure S12 a) ESR spectra of CWO after various treatments. b) Comparison of SOSG after different treatments,


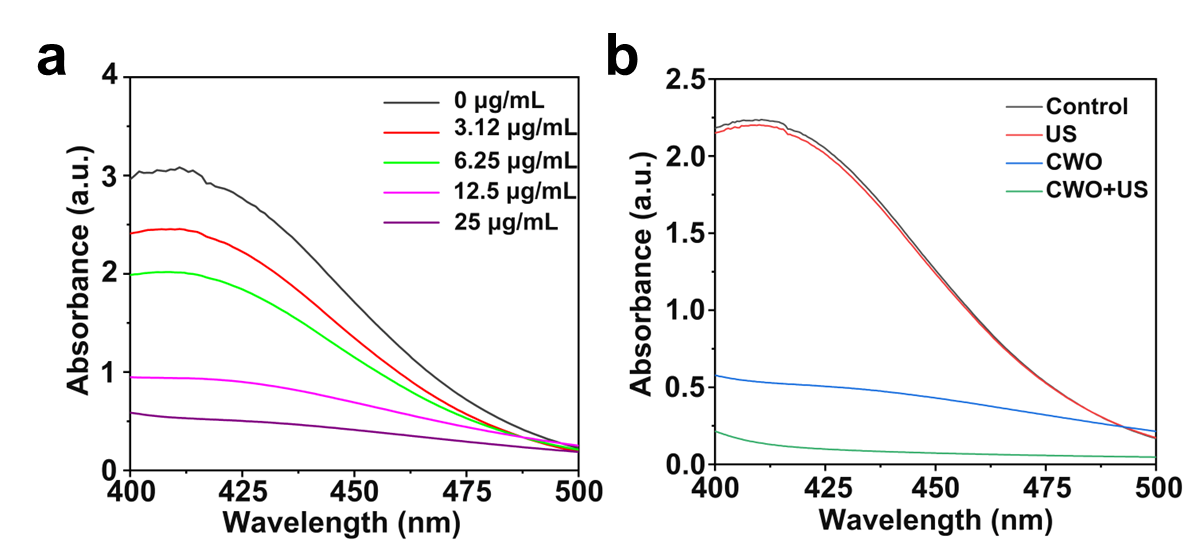


Figure S13 a) GSH consumption with different concentration of CWO. b) Comparison of GSH consumption after different treatments, US irradiation for 3 min.


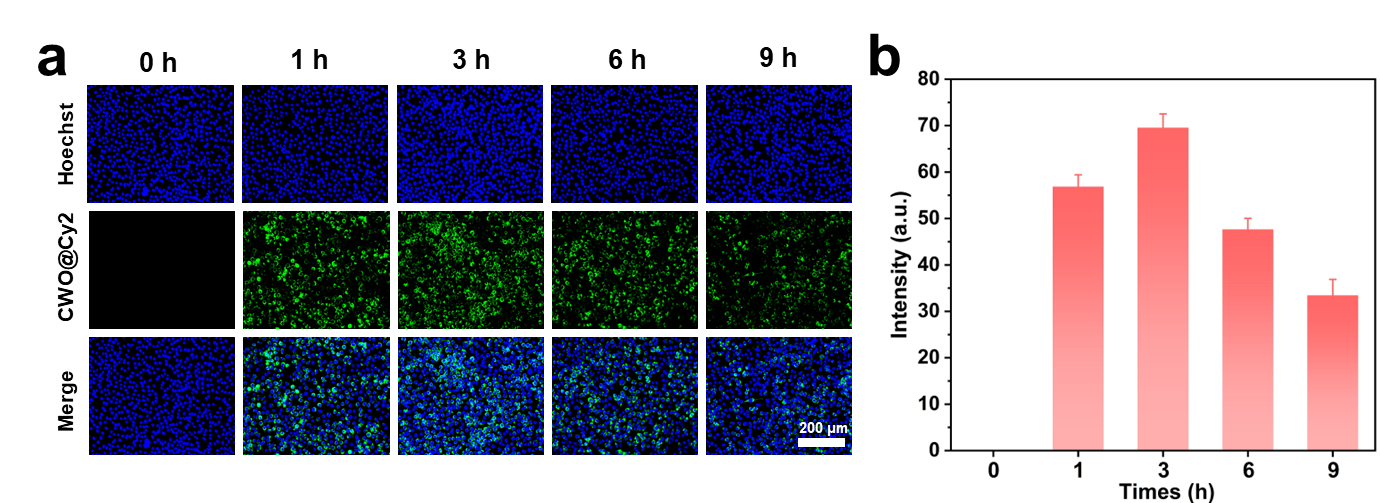


Figure S14 a) Fluorescence images of Panc02 cells treated with CWO@Cy2. b) The relative fluorescence intensity at different times.


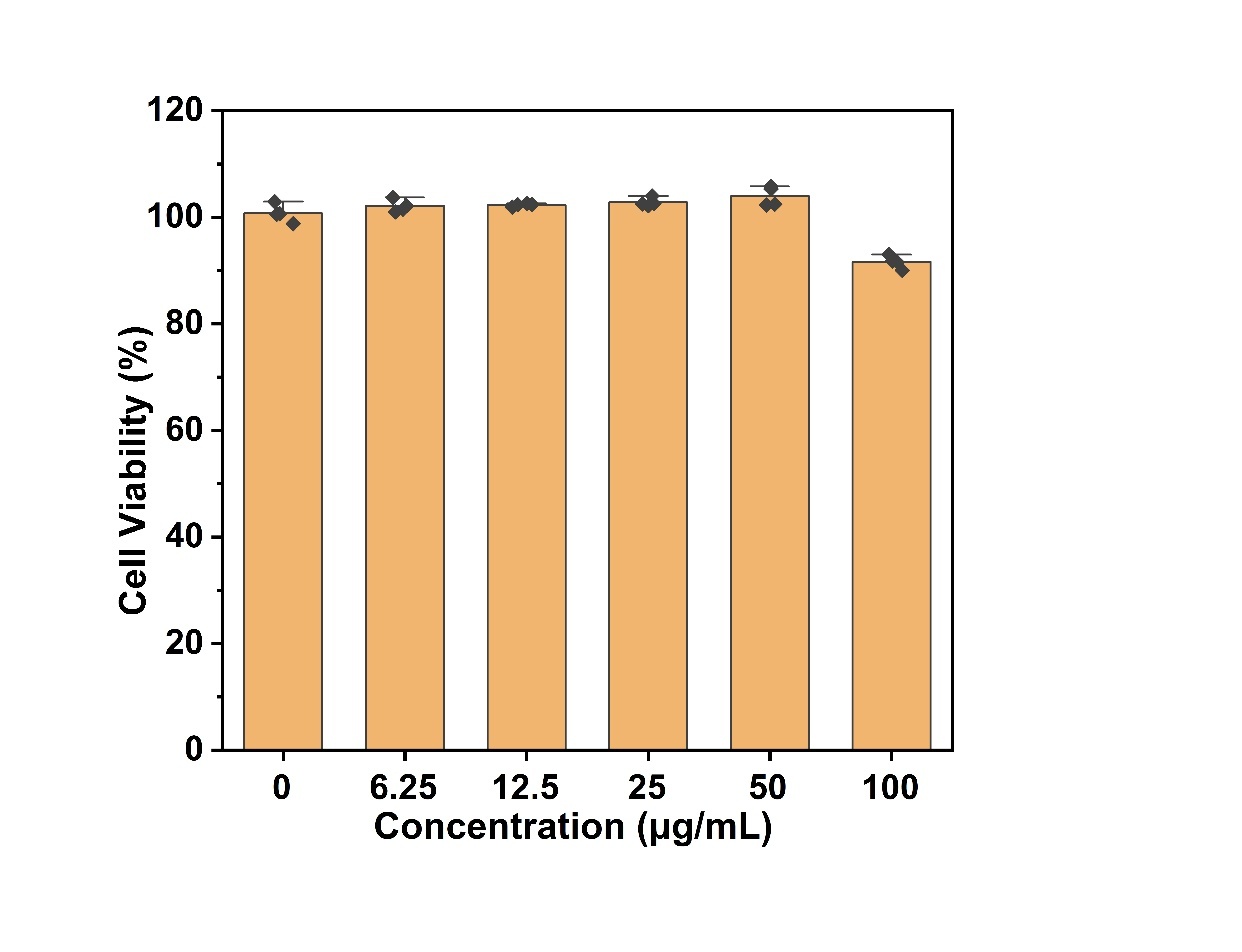


Figure S15 CWO cytotoxicity in Panc02 cells.


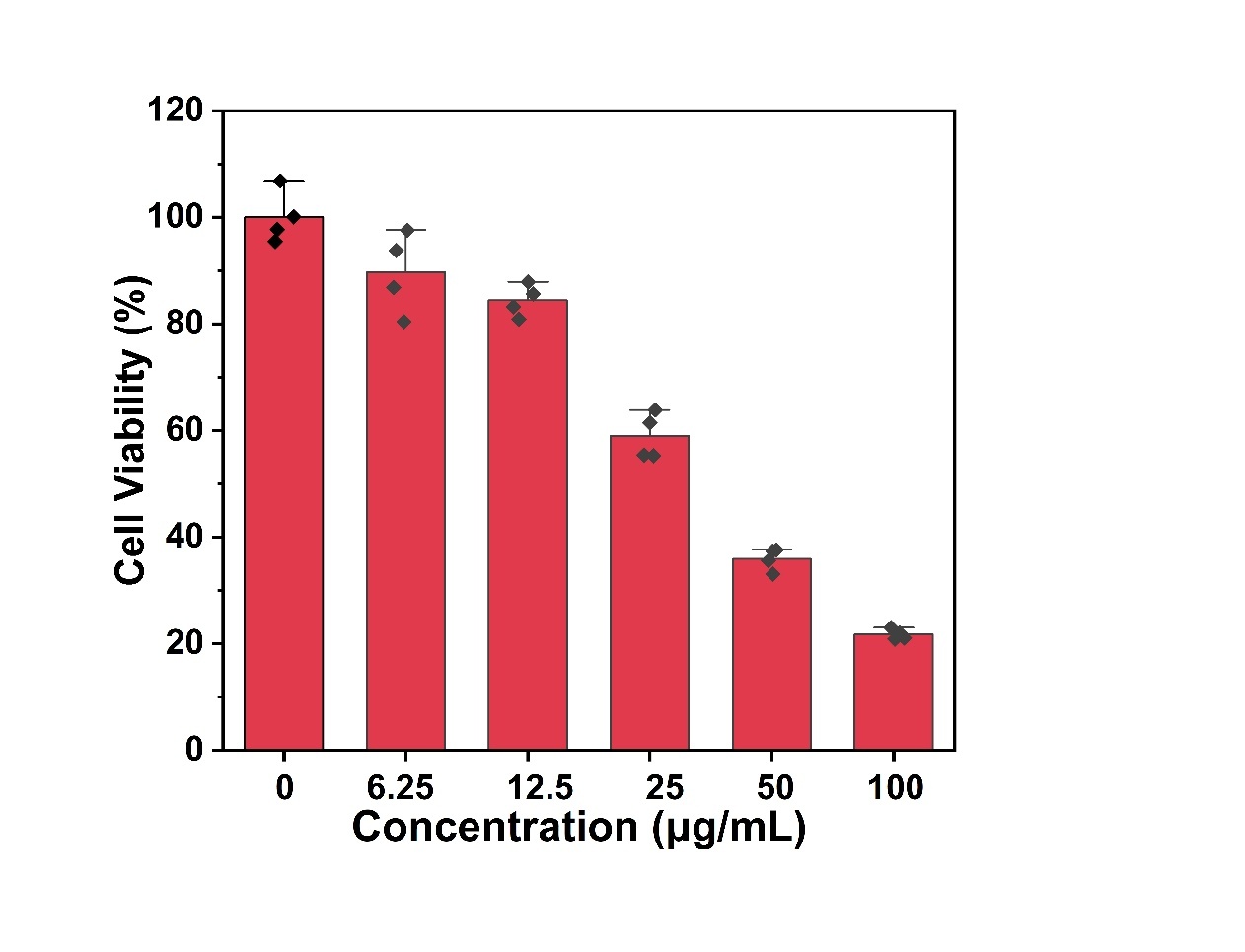


Figure S16 Therapeutic efficiency of CWO at different concentrations in Panc02 cells.


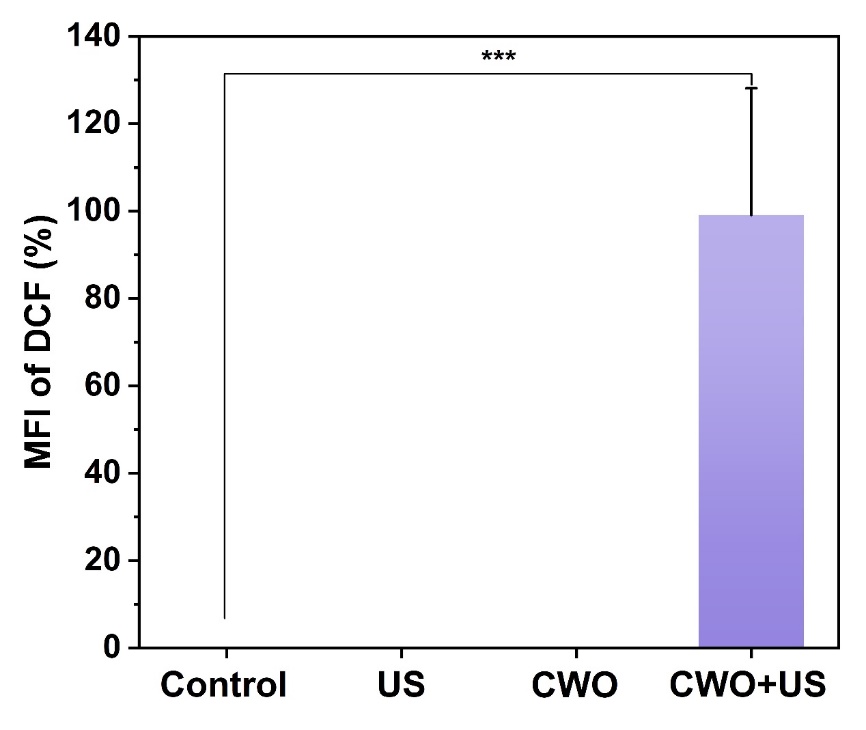


Figure S17 Corresponding quantification of mean fluorescence intensity of DCF inside cells (n=3). Statistical analysis was performed by one-way ANOVA. ***P < 0.001.


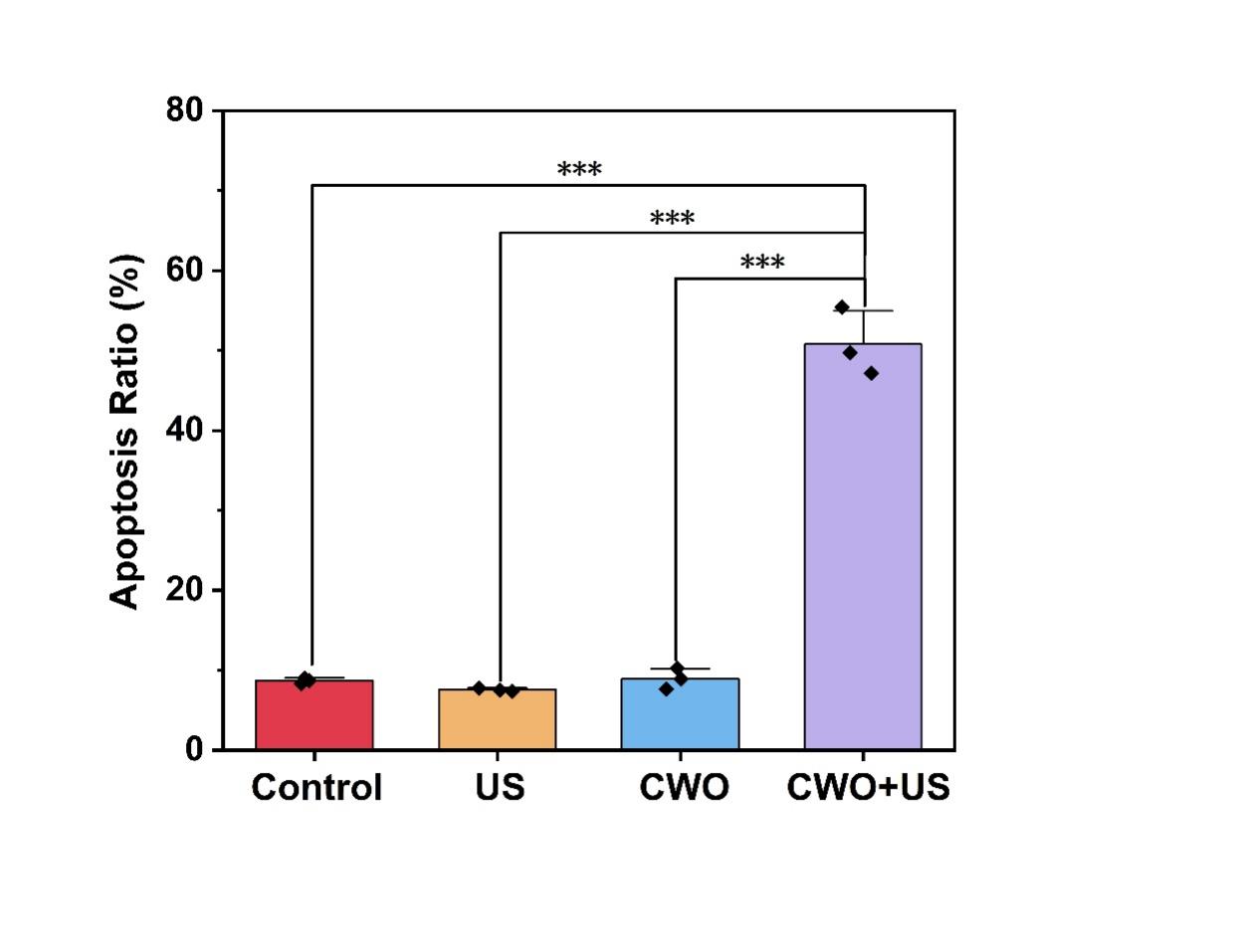


Figure S18 Corresponding quantification of apoptosis rate from the flow cytometry results (n=3). Statistical analysis was performed by one-way ANOVA. ***P < 0.001.


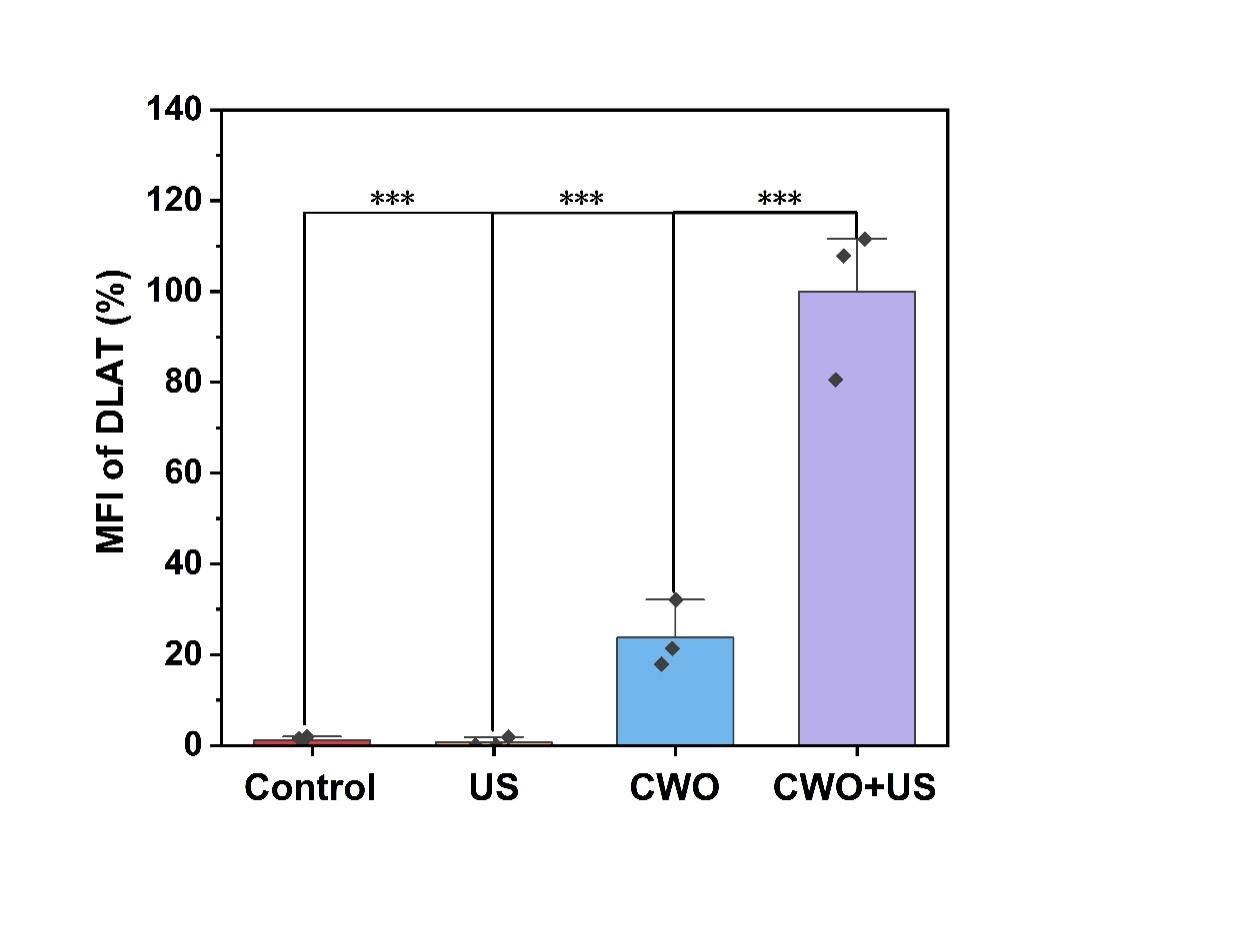


Figure S19 Corresponding quantification of mean fluorescence intensity of DLAT inside cells (n=3). Statistical analysis was performed by one-way ANOVA. ***P < 0.001.


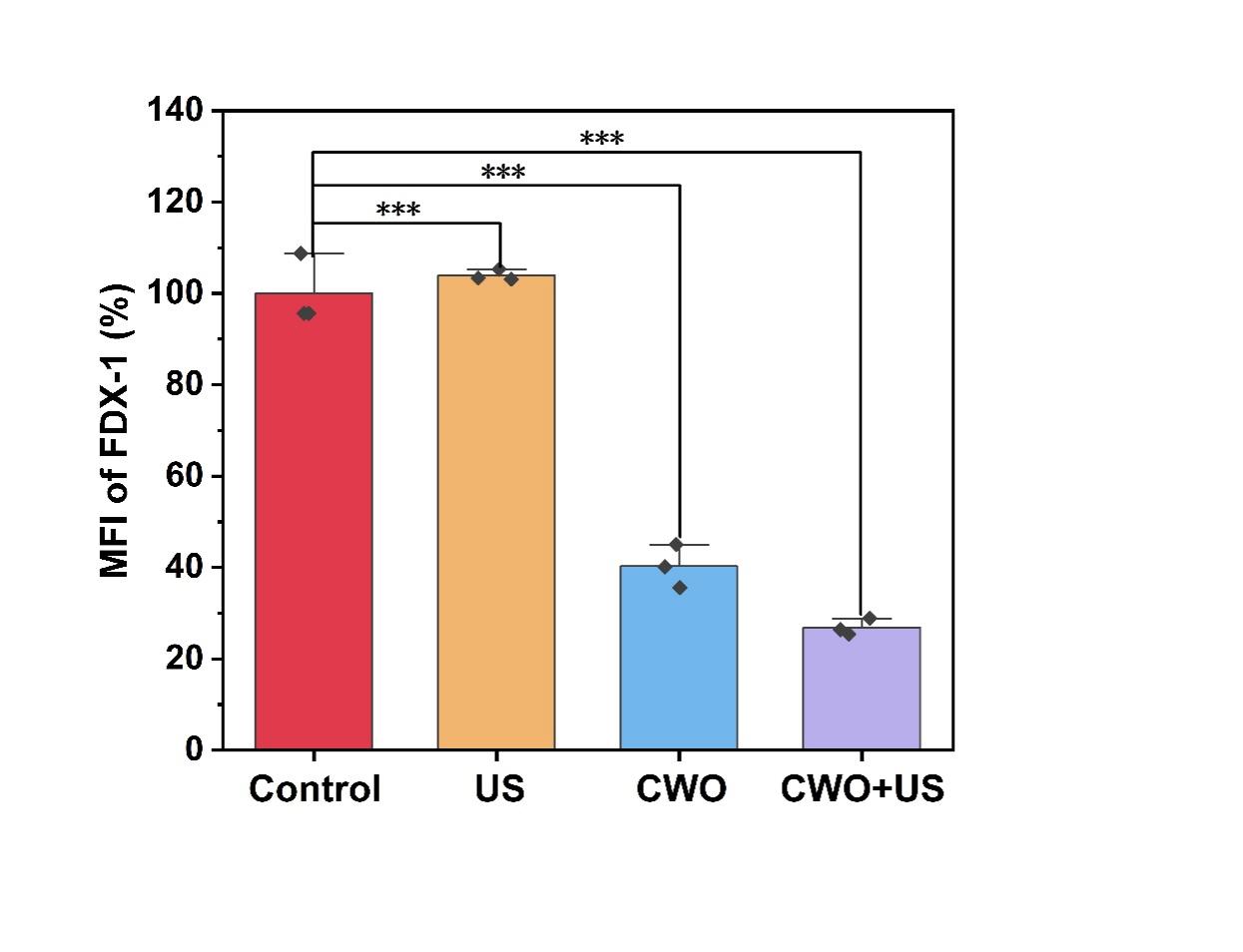


Figure S20 Corresponding quantification of mean fluorescence intensity of FDX-1 inside cells (n=3). Statistical analysis was performed by one-way ANOVA. ***P < 0.001.


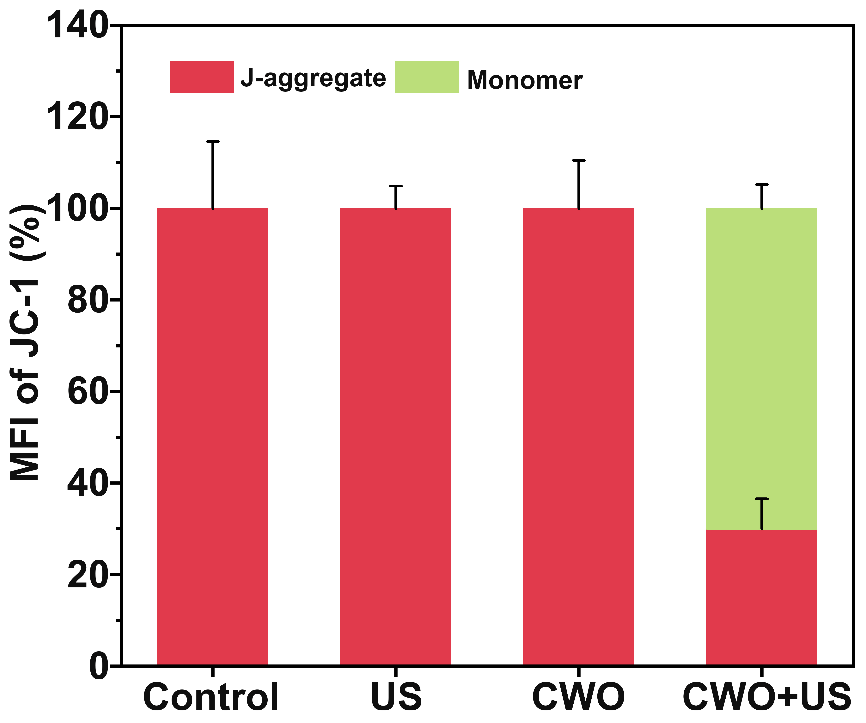


Figure S21 Corresponding quantification of mean fluorescence intensity of JC-1 inside cells (n=3).


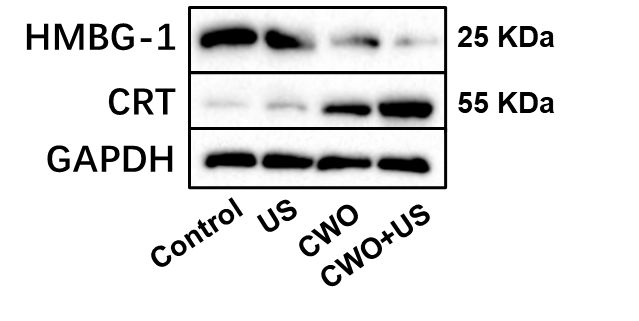


Figure S22 Western blot analysis on the expressions of HMGB-1and CRT.


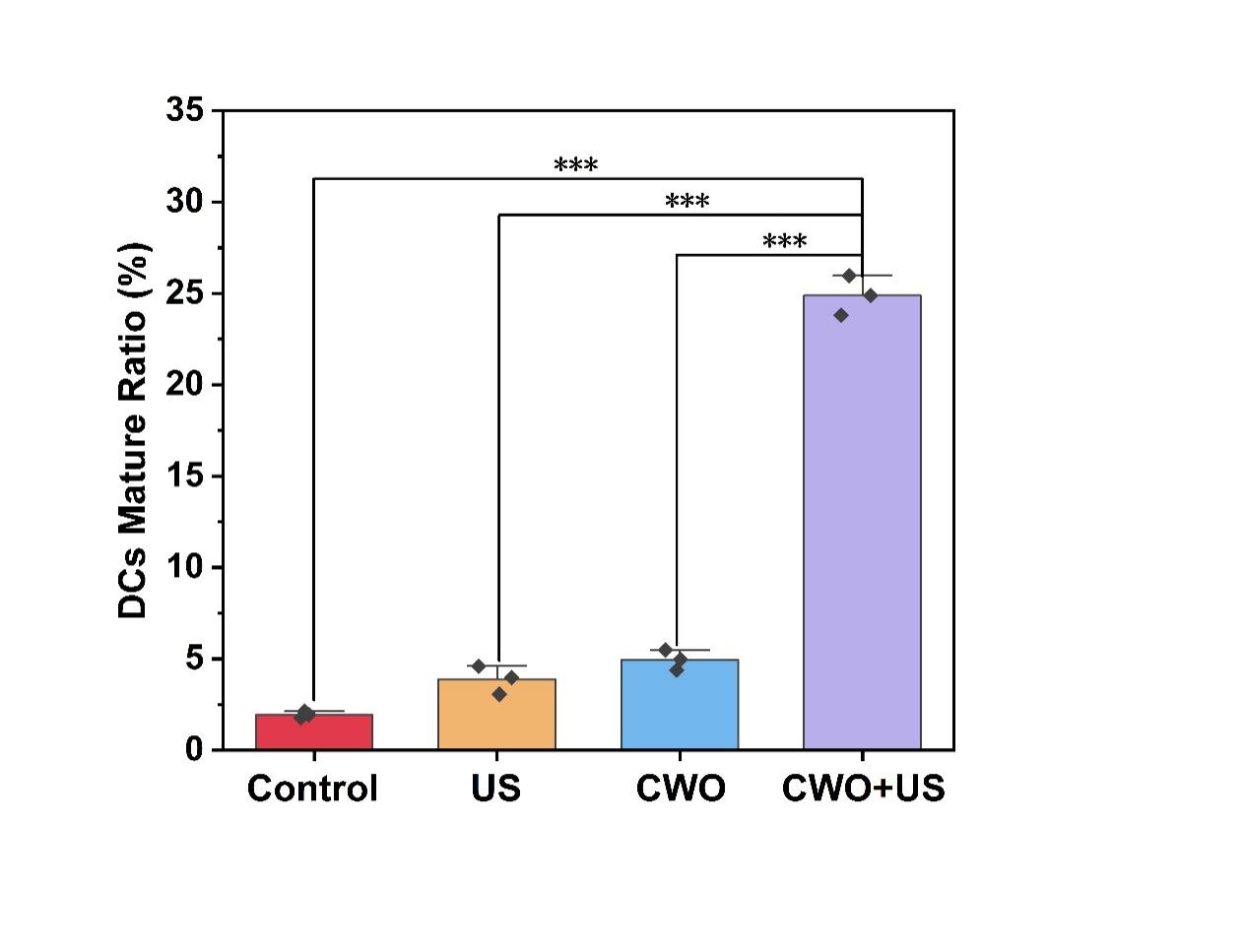


Figure S23 Corresponding quantification of DCs maturation rate from the flow cytometry results (n=3). Statistical analysis was performed by one-way ANOVA. ***P < 0.001.


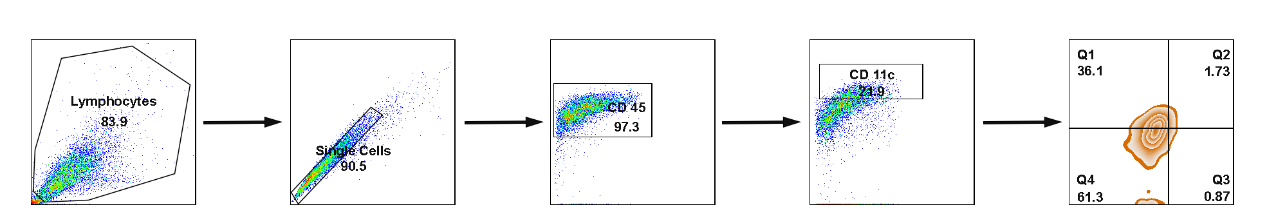


Figure S24 The gating strategy used to determine the percentage of matured DCs (CD11c^+^ CD80^+^ CD86^+^).


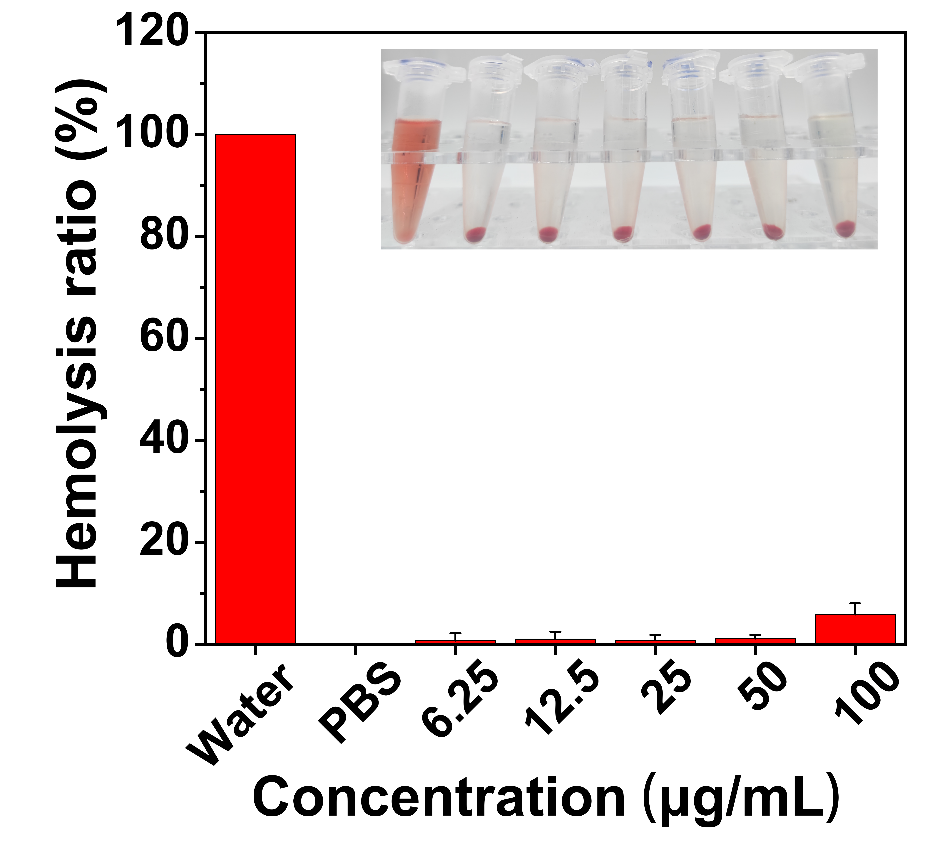


Figure S25 Hemolysis of CWO with different concentrations.


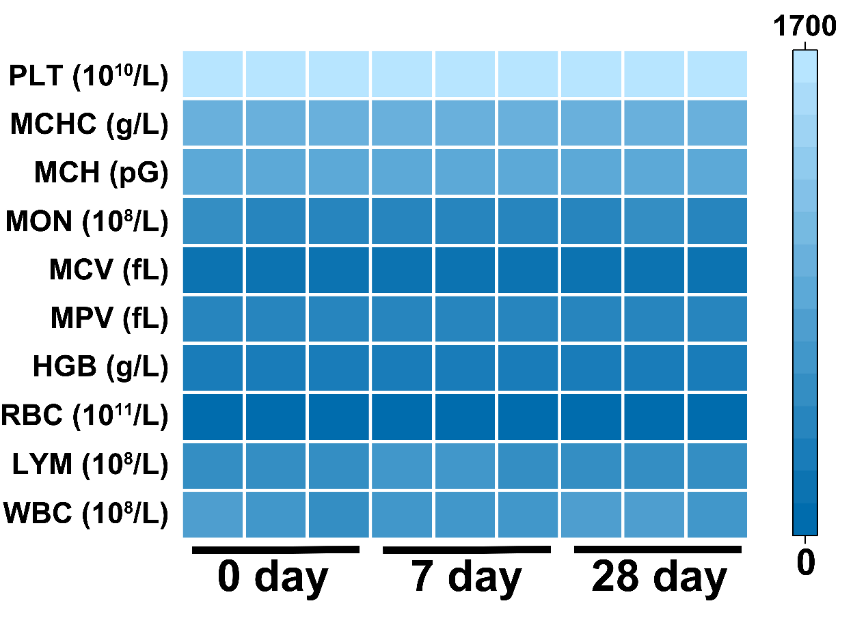


Figure S26 The hematology indexes of BALB/c mice treated with CWO (15 mg kg^–1^) at different times (0, 7, and 28 days).


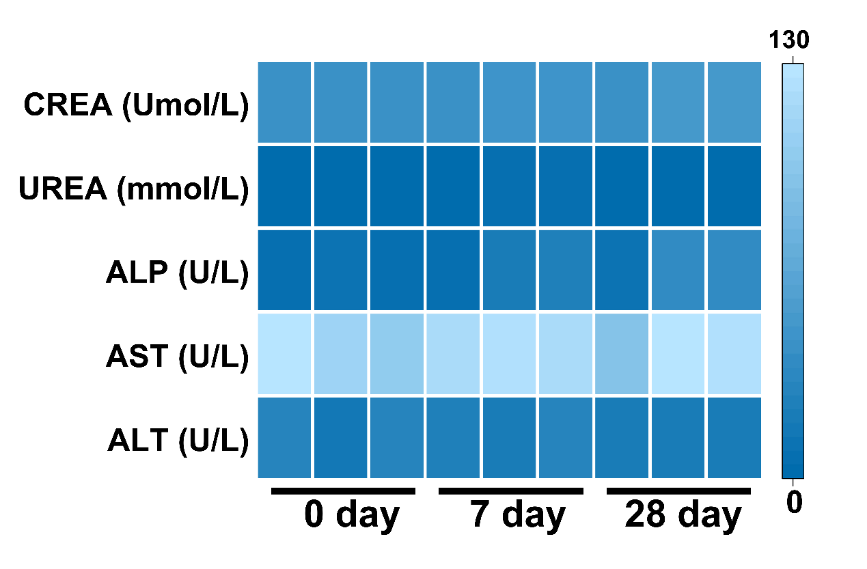


Figure S27 The blood biochemistry indexes of BALB/c mice treated with CWO (15 mg kg^–1^) at different times (0, 7, and 28 days).


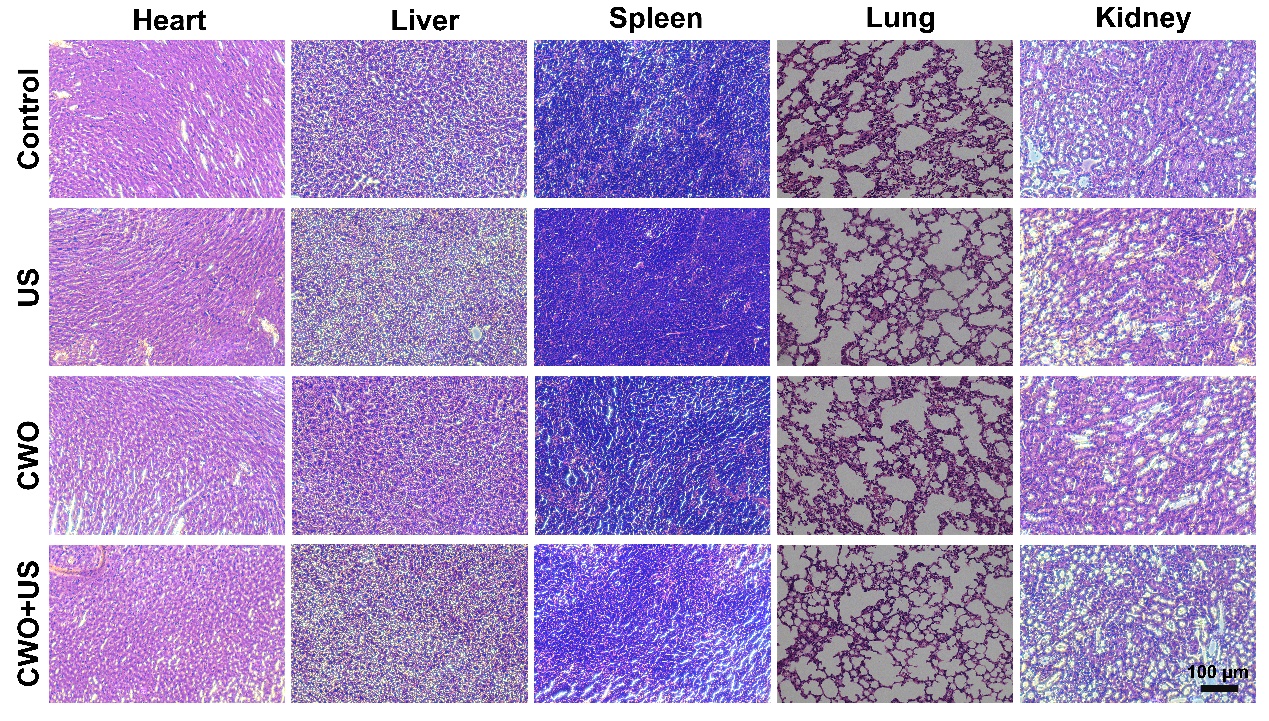


Figure S28 H&E staining of heart, liver, spleen, lung, and kidney after different treatment.


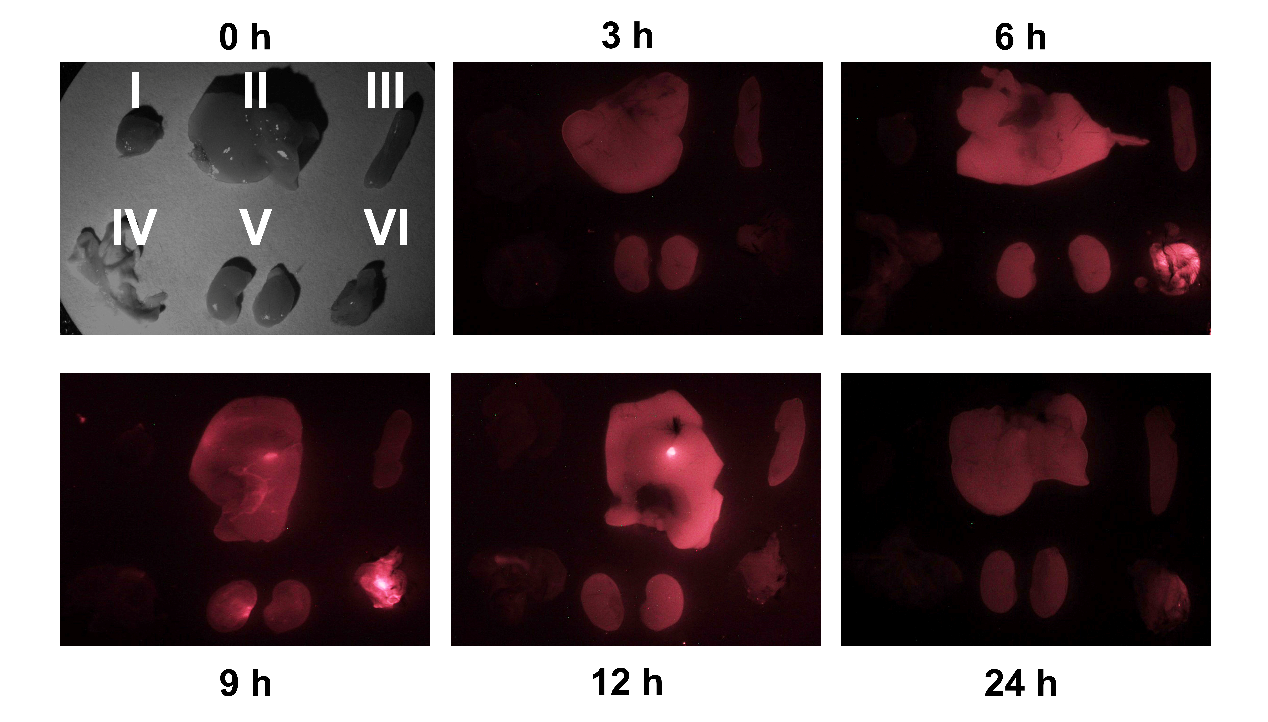


Figure S29 The *ex vivo* fluorescence image of tumor and major organs with intravenous injection of CWO@Cy5.5 recorded at 0, 3, 6, 9, 12 and 24 h. I: heart; II: liver; III: spleen; IV: lung; V: kidney; VI: tumor.


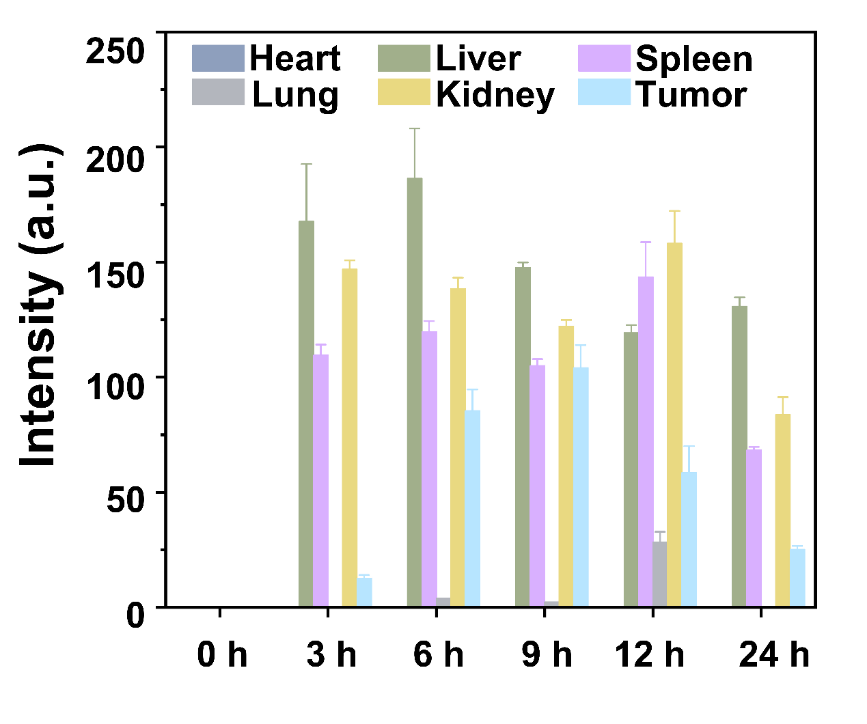


Figure S30 Corresponding quantification of mean fluorescence intensity of tumor and major organs. Values are expressed as mean ± SD (n = 3).


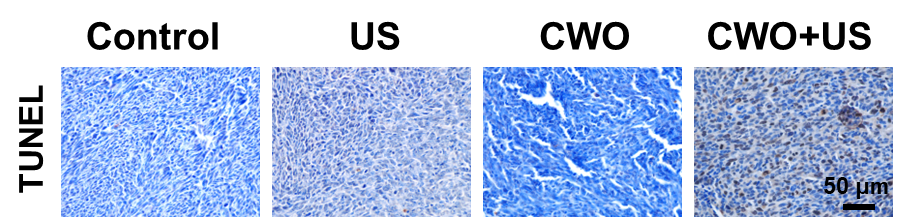


Figure S31 TUNEL stained tumor slices in different groups.


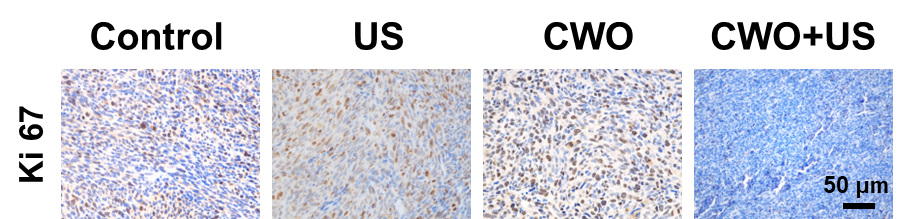


Figure S32 Ki 67 stained tumor slices in different groups.

Table S1. Representative piezoelectric sonosensitizers.

| **Paradigm** | **Bandgap** | **References** |
| --- | --- | --- |
| FX11@TPEG-WS_2_ | 2.30 eV | 1 |
| Mg-HAP@MS/ONC201 | 5.30 eV | 2 |
| Mn-ZnO | 2.95 eV | 3 |
| ATO@Bi-HJ | 2.75 eV | 4 |
| G-Bi_2_MoO_6_ | 2.97 eV | 5 |
| Au-ZnO | 2.95 eV | 6 |
| PCBO | 1.83 eV | 7 |
| CWO | 1.71 eV | This work |

**References**

1. Q. Hoang, K. Huynh, T. Cao, J. Kang, X. Dang, V. Ravichandran, H. Kang, M. Lee, J. Kim, Y. Ko, T. Lee, M. Shim, *Adv. Mater*. **2023**, 35, 2300437.
2. J. Yang, Y. Du, Y. Yao, Y. Liao, B. Wang, X. Yu, K. Yuan, Y. Zhang, F. He, P. Yang, *Adv. Sci*. **2024**, 11, 2307130.
3. B. Tian, R. Tian, S. Liu, Y. Wang, S. Gai, Y. Xie, D. Yang, F. He, P. Yang, J. Lin, *Adv. Mater.* **2023**, 35, 2304262.
4. X. Cao, Y. Wang, X. Song, W. Lou, X. Li, W. Lu, K. Chen, L. Chen, Y. Chen, B. Huang, *Adv. Funct. Mater*. **2023**, 33, 2300777.
5. Y. Dong, S. Dong, B. Liu, C. Yu, J. Liu, D. Yang, P. Yang, J. Lin, *Adv. Mater*. **2021**, 33, 2106838.
6. J. Cheng, W. Pan, Y. Zheng, J. Zhang, L. Chen, H. Huang, Y. Chen, R. Wu, *Adv. Mater.* **2024**, 2312102.
7. J. Wang, H. Zheng, G. Hu, X. Yang, H. You, L. Dong, S. Song, *Adv. Sci.* **2024**, 4, 2402599.
